# Efficacy and safety of PD-1/PD-L1 inhibitors in advanced or recurrent endometrial cancer: a meta-analysis with trial sequential analysis of randomized controlled trials

**DOI:** 10.3389/fimmu.2025.1521362

**Published:** 2025-01-31

**Authors:** Ji Ren, Jinghe Wang, Yanan Wang, Dongying Yang, Jianming Sheng, Shili Zhu, Yunli Liu, Xiaoqi Li, Wei Liu, Binbin Zhang

**Affiliations:** ^1^ Department of Medicine and Health, Dezhou University, Dezhou, China; ^2^ Department of General Surgery, Qilu Hospital of Shandong University, Jinan, China

**Keywords:** PD-1 inhibitors, PD-L1 inhibitors, immune checkpoint inhibitors, chemotherapy, endometrial cancer

## Abstract

**Background:**

The combination of programmed cell death 1 (PD-1)/programmed death ligand 1 (PD-L1) inhibitors with chemotherapy (CT) is currently under evaluation as a first-line treatment for advanced or recurrent endometrial cancer (EC). This study sought to assess the efficacy and safety of this therapeutic combination in patients with advanced or recurrent EC.

**Methods:**

We performed an exhaustive review of randomized controlled trials (RCTs) up to September 25, 2024, examining the efficacy and safety of combining PD-1/PD-L1 inhibitors with CT versus CT alone (or plus placebo) in advanced or recurrent EC. Efficacy was measured by progression-free survival (PFS) and overall survival (OS), while safety was assessed by the incidence of any grade or grade ≥ 3 adverse events (AEs). We calculated hazard ratios (HRs) for PFS and OS, as well as risk ratios (RRs) for AEs, each accompanied by 95% confidence intervals (CIs). To evaluate heterogeneity, we employed Cochran’s Q test, I^2^ statistics, and 95% prediction intervals (PIs). Trial sequential analysis (TSA) was conducted using R Version 4.3.1, STATA Version 12.0, and TSA Version 0.9.5.10 Beta software.

**Results:**

Our analysis incorporated 6 studies, encompassing a total of 2,954 patients. The combination of PD-1/PD-L1 inhibitors with CT significantly improved PFS (HR = 0.617, 95% CI: 0.506-0.752; 95% PI: 0.334-1.140) and OS (HR = 0.774, 95% CI: 0.664-0.902; 95% PI: 0.553-1.083) compared to CT alone (or plus placebo) in the overall population. Subgroup analysis based on mismatch repair (MMR) status revealed pronounced benefits in PFS and OS for patients with deficient MMR (dMMR) (PFS: HR = 0.344, 95% CI: 0.269-0.438; 95% PI: 0.231-0.510; OS: HR = 0.371, 95% CI: 0.245-0.562; 95% PI: 0.025-5.461) compared to those with proficient MMR (pMMR) (PFS: HR = 0.772, 95% CI: 0.627-0.950; 95% PI: 0.394-1.512; OS: HR = 0.996, 95% CI: 0.692-1.435; 95% PI: 0.021-47.662). Although there was no observed difference in the incidence of any grades AEs (RR = 0.994, 95% CI: 0.982-1.006; 95% PI: 0.978-1.009), the risk of grade ≥ 3 AEs was elevated in the group receiving PD-1/PD-L1 inhibitors in combination with CT (RR = 1.132, 95% CI: 1.023-1.252; 95% PI: 0.836-1.532).

**Conclusion:**

The combination of PD-1/PD-L1 inhibitors with CT significantly improved PFS and OS in advanced or recurrent EC patients, with particularly pronounced benefits observed in those with dMMR. Clinicians can tailor treatment strategies according to individual patient characteristics to optimize therapeutic outcomes, while remaining alert to the possibility of AEs in clinical practice.

**Systematic review registration:**

https://www.crd.york.ac.uk/PROSPERO/, identifier CRD42024595455.

## 1 Introduction

Endometrial cancer (EC) ranks as the second most prevalent gynecological malignancy worldwide, with both its incidence and mortality rates on the rise (1–3). Traditionally, carboplatin-paclitaxel chemotherapy (CT) has been the standard first-line treatment for advanced or recurrent EC. However, the prognosis remains dismal, with a median overall survival (OS) of less than three years (4, 5). There is an urgent need for novel therapeutic approaches to prevent recurrence and extend patient survival. Recent investigations have identified immune checkpoint inhibitors (ICIs) as a promising treatment option for advanced or recurrent EC (6–9). Approximately 25-30% of EC cases exhibit deficient mismatch repair (dMMR) and high microsatellite instability (MSI-H) (10, 11). The elevated expression of the programmed cell death 1 (PD-1) receptor and its ligands, programmed death ligand 1 (PD-L1), associated with the high mutational burden in dMMR/MSI-H EC, renders this subtype particularly responsive to ICIs, especially anti-PD-1 and anti-PD-L1 agents (12–14).

In 2017, Ott and colleagues first conducted an assessment of the impact of PD-1/PD-L1 inhibitors on individuals with advanced or recurrent EC characterized by dMMR and proficient mismatch repair (pMMR). Their findings revealed a remarkable 100% objective response rate (ORR) in dMMR patients, whereas those with pMMR exhibited an ORR of merely 5.6% (15). For patients with advanced disease that has progressed following platinum-based CT, monotherapy with PD-1 inhibitor dostarlimab or pembrolizumab is currently established as the standard treatment for the dMMR/MSI-H subgroup (16). Recent progress has been made in treating primary advanced or recurrent EC through combination therapies that integrate PD-1/PD-L1 inhibitors with CT (17–20). While it is evident that dMMR patients derive significant benefit from adding PD-1/PD-L1 inhibitors to CT, the advantage for pMMR patients remains uncertain (17, 20). Additionally, in two clinical trials, the addition of PD-L1 inhibitor avelumab and atezolizumab did not show improvements in progression-free survival (PFS) and OS (17, 20). Furthermore, the distinct mechanisms of action exhibited by anti-PD-1 and anti-PD-L1 agents, as demonstrated in other solid tumors, might explain the varying effectiveness of immunotherapy in the pMMR population (21). Although pMMR patients constitute a heterogeneous subgroup, necessitating further molecular subclassifications and the development of targeted therapies, the effectiveness of immunotherapy in this population remains to be elucidated.

Recent years have seen some pooled analyses investigating the effects of ICIs combined with CT on advanced or recurrent EC, specifically in patients with dMMR or pMMR status (16, 22, 23). With the publication of new follow-up results from several high-quality randomized controlled trials (RCTs) (24–26), it becomes imperative to incorporate these findings into a comprehensive review. In addition, these trials encompassed a range of patient characteristics, such as MMR status, age, ethnicity, disease progression, histology category, and PD-1/PD-L1 expression, resulting in variability in adverse events (AEs) and survival outcomes among different subgroups. Consequently, we aimed to conduct a meta-analysis of RCTs to evaluate the potential efficacy and safety benefits of PD-1/PD-L1 inhibitors in combination with standard CT, compared to CT alone or plus placebo, in patients with advanced or recurrent EC.

## 2 Materials and methods

### 2.1 Study design

This meta-analysis has been registered with the International Prospective Register of Systematic Reviews (PROSPERO, CRD42024595455) and was conducted in accordance with the guidelines set forth by the Preferred Reporting Items for Systematic Reviews and Meta-Analyses (PRISMA) (27).

### 2.2 Search strategy

A comprehensive and systematic search was conducted across the PubMed, Web of Science, Embase, and Cochrane Library electronic databases to obtain an initial list of pertinent studies. This literature search encompassed all records from their inception until September 25, 2024, without any language restrictions. The search utilized the following terms: (“immune checkpoint inhibitor” OR “ICI” OR “programmed cell death protein 1 inhibitor” OR “programmed death-ligand 1 inhibitor” OR “PD-1” OR “PD-L1” OR “pembrolizumab” OR “dostarlimab” OR “durvalumab” OR “atezolizumab” OR “avelumab” OR “nivolumab”) AND (“endometrial cancer” OR “endometrial neoplasms” OR “endometrial carcinoma” OR “cancer of endometrium”). Detailed search strategy for each database was provided in **Supplementary File 1**. Additionally, we manually reviewed the reference lists of the selected review articles to identify any additional studies suitable for inclusion in our meta-analysis.

### 2.3 Inclusion and exclusion criteria

Eligible studies were identified based on the following criteria: (1) RCTs; (2) participants were adult females with a diagnosis of advanced or recurrent EC; (3) the treatment regimen involved PD-1/PD-L1 inhibitors combined with CT, followed by maintenance therapy with PD-1/PD-L1 inhibitors; (4) the comparison group received CT with or without a placebo, followed by either placebo maintenance or no maintenance therapy; (5) the primary outcome was the hazard ratio (HR) for PFS or OS, while secondary outcomes included any grade AEs or grade ≥ 3 AEs. Exclusion criteria encompassed: (1) Studies that were single-arm, non-randomized, or non-interventional; (2) publications lacking data on survival and safety outcomes or containing duplicate information; (3) research involving single-agent PD-1/PD-L1 inhibitors or combinations with multiple tyrosine kinase inhibitors (TKIs) or poly (ADP-ribose) polymerase (PARP) inhibitors; (4) conference abstracts, review articles, study protocols, case reports, or letters.

### 2.4 Data extraction and risk of bias assessment

Two independent reviewers extracted data from the qualifying studies and recorded it using a standardized template. The extracted information included the first author’s name, publication year, trial designation, study phase, region, criteria for eligible EC patients, sample size, participant age, treatment regimens for both experimental and control groups, MMR status, and follow-up duration. In cases where multiple reports were available for the same RCT, the most recent or detailed publication was selected to ensure the inclusion of the most complete and up-to-date data. If direct reports of PFS or OS were not provided, we employed Engauge Digitizer Version 10.8 software in conjunction with the method developed by Tierney et al. (28) to estimate these values from Kaplan-Meier curves (29).

Two investigators independently conducted the risk of bias assessment for RCTs utilizing the modified Jadad scale (30). This scale comprises five criteria for RCT evaluation, assigning scores from 0 to 7 based on randomization, allocation concealment, blinding, and dropout/withdrawal. Scores from 0 to 3 denote low quality, while scores of 4 or higher represent high quality. Any discrepancies in quality assessment were resolved through consensus discussions with a third reviewer.

### 2.5 Statistical analysis

Statistical analyses were executed using R Version 4.3.1 and STATA Version 12.0. HRs were employed to evaluate PFS and OS, with HRs greater than 1 indicating a benefit for the control group, and HRs less than 1 indicating a benefit for the intervention group. Binary outcomes were analyzed using risk ratios (RRs) with 95% confidence intervals (CIs). Study heterogeneity was assessed using Cochran’s Q test, I^2^ statistics, and 95% prediction intervals (PIs) (31, 32), with I^2^ values of 25%, 50%, and 75% representing low, moderate, and high heterogeneity, respectively (33). A fixed-effects model was applied when heterogeneity was low; otherwise, a random-effects model was employed (34). Subgroup analyses for PFS and OS were conducted based on stratified results from the included RCTs or the types of ICIs (PD-1 or PD-L1 inhibitors). Sensitivity analyses were performed by sequentially excluding individual studies and recalculating the combined effect sizes to assess the robustness of the overall findings. Publication bias was assessed using funnel plots and Begg’s and Egger’s tests (35, 36). A two-sided *p*-value of less than 0.05 was considered indicative of statistical significance.

### 2.6 Trial sequential analysis

Trial sequential analysis (TSA) was employed in the meta-analysis to mitigate the risk of false-positive or false-negative results (37). This methodology was implemented using TSA Version 0.9.5.10 Beta for binary outcomes. For PFS and OS, TSA was performed using STATA Version 12.0 and R Version 4.3.1, applying the *a priori* information size (APIS) method. If the cumulative Z-curve intersected the trial sequential monitoring boundary or entered the futility zone, sufficient evidence for the expected intervention effect was established, indicating no further studies were necessary. Conversely, if the Z-curve failed to intersect any boundaries or if the required information size (RIS) or APIS was not achieved, the evidence was deemed inadequate, necessitating additional trials to substantiate the results. In conducting TSA, a two-sided α of 0.05, a power (1-β) of 0.90, and a 15% RR reduction were used to determine the RIS and APIS. The control event proportion was derived from the comparator group.

## 3 Results

### 3.1 Study selection

**Figure 1:**
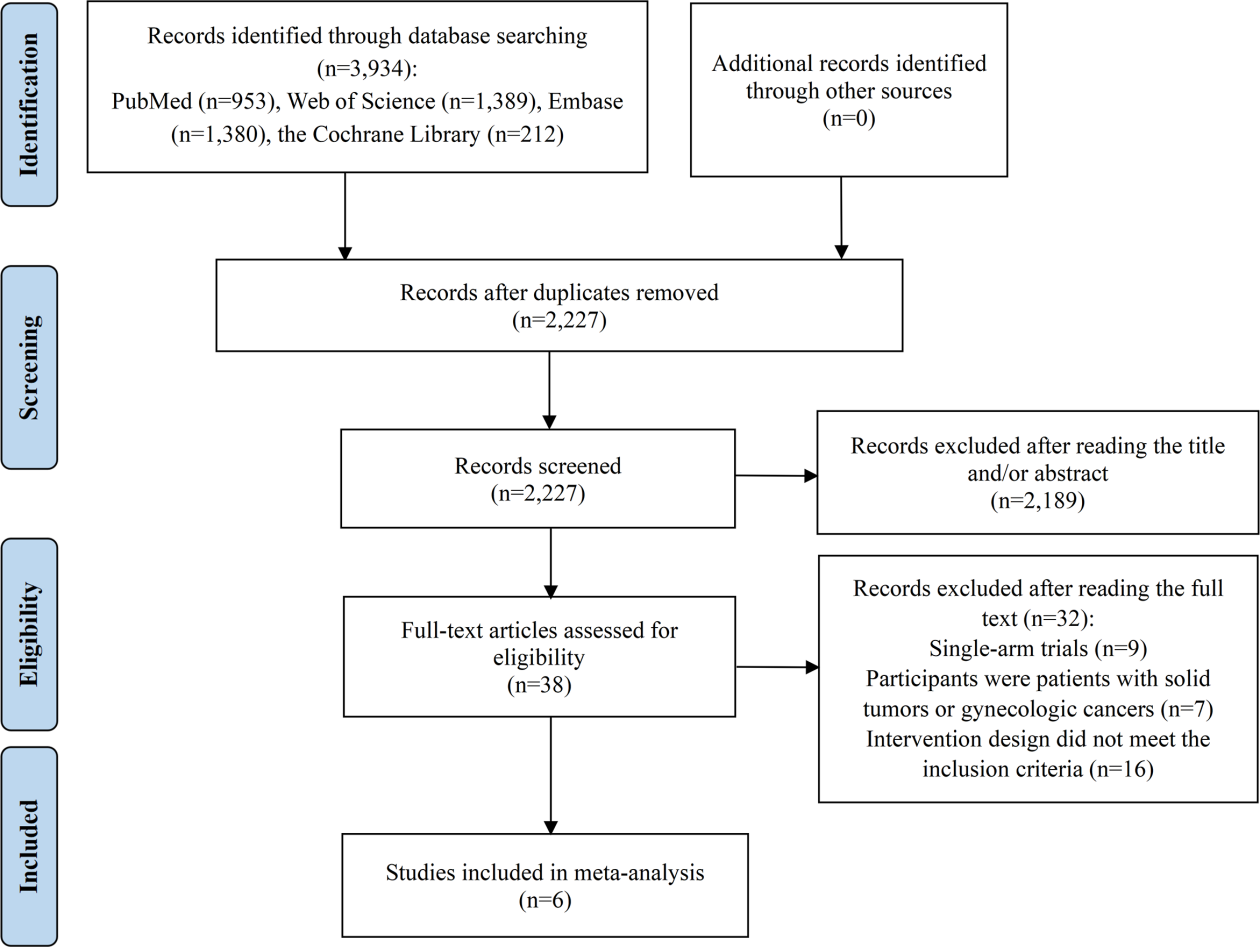
Flow diagram of the process of study selection.

The selection process is depicted in a PRISMA flow diagram (**Figure 1**). An initial search across all databases identified 3,934 potentially relevant records. After removing duplicates, 2,227 articles remained for title and abstract screening. Of these, 2,189 were excluded as they did not meet the relevance criteria, leaving 38 articles for an in-depth full-text review to determine their eligibility for inclusion. Upon detailed evaluation, 32 studies were excluded for the following reasons: 9 were single-arm trials, 7 involved patients with solid tumors or gynecologic cancers, and 16 had intervention group treatment regimens that did not meet the inclusion criteria. Consequently, 6 studies were incorporated into this meta-analysis (17–19, 24–26).

### 3.2 Characteristics and quality assessment of selected studies

The detailed characteristics of the studies included in this meta-analysis were summarized in **Table 1**. This meta-analysis encompassed 6 studies involving 5 RCTs, with 4 being phase III and 1 phase II. Both Mirza et al. (19) and Powell et al. (25) provided distinct findings from the RUBY trial at various follow-up periods, warranting the inclusion of both reports. Overall, 1,556 EC patients were assigned to receive PD-1/PD-L1 inhibitors in combination with carboplatin-paclitaxel CT, while 1,398 patients were assigned to carboplatin-paclitaxel CT alone or plus a placebo. Among these EC patients, 2,197 (74.4%) exhibited pMMR, 738 (25.0%) had dMMR, and 19 (0.6%) had undetermined MMR status. The PD-1/PD-L1 inhibitors administered included pembrolizumab, dostarlimab, durvalumab, avelumab, and atezolizumab. The 5 RCTs were deemed high-quality due to rigorous trial designs that accounted for factors like randomization, allocation concealment, blinding, and handling of withdrawals and dropouts, and they were published in journals of considerable impact. Notably, the MITO END-3 trial was open-label and did not employ a double-blind design (17), resulting in a slightly lower quality assessment compared to the other trials (**Supplementary File 2**).

**Table 1:** Summary of the characteristics of included RCTs.

Study (first author, year)	Trial name	Study phase	Region	Participants	Sample size (I/C)	Age (I/C, year)	Experimental group	Control group	MMR status			Follow-up time (month, median)
									dMMR	pMMR	NR	
Eskander et al., 2023 (18)	NRG-GY018	Phase 3	395 sites in four countries (the United States, Canada, Japan, and South Korea)	Adult women (≥18 years of age) with confirmed advanced-stage, metastatic, or recurrent endometrial cancer of any histologic subtype except for carcinosarcoma; stage III or IVA according to the RECIST; ECOG PS of 0-2	dMMR: 112/113; pMMR: 293/295	dMMR: Median (range): 66 (37-85); pMMR: Median (range): 65.5 (29-93)	Pembrolizumab + paclitaxel-carboplatin (6 cycles) followed by pembrolizumab maintenance (up to 14 cycles)	Placebo + paclitaxel-carboplatin (6 cycles) followed by placebo maintenance (up to 14 cycles)	225	588	0	dMMR: 12; pMMR: 7.9
Powell et al., 2024 (25)	RUBY	Phase 3	113 sites in 19 countries	Patients (≥ 18 years of age) with histologically or cytologically confirmed primary advanced (FIGO stage III/IV) or recurrent EC; stage IIIA, IIIB, or IIIC1 according to the RECIST	245/249	Median (range): 64 (41-81)/65 (28-85)	Dostarlimab + paclitaxel-carboplatin (6 cycles) followed by dostarlimab maintenance (up to 3 years)	Placebo + paclitaxel-carboplatin (6 cycles) followed by placebo maintenance (up to 3 years)	118	376	0	37.2 (range 31.0-49.5)
Westin et al., 2024 (26)	ENGOT-EN10	Phase 3	22 countries	Patients (age 18 years and older) with newly diagnosed advanced (FIGO stage III/newly diagnosed stage IV) or recurrent endometrial cancer of epithelial histology (excluding sarcomas)	238/241	Median (range): 64 (22-84)/64 (31-85)	Durvalumab + paclitaxel-carboplatin (6 cycles) followed by durvalumab maintenance (every 4 weeks)	Placebo + paclitaxel-carboplatin (6 cycles) followed by placebo maintenance (every 4 weeks)	95	384	0	I: 18.4 (range 2.1-33.0); C: 18.6 (range 0.5-32.9)
Mirza et al., 2023 (19)	RUBY	Phase 3	113 sites in 19 countries	Patients (≥ 18 years of age) with histologically or cytologically confirmed primary advanced (FIGO stage III/IV) or recurrent EC; stage IIIA, IIIB, or IIIC1 according to the RECIST	245/249	Median (range): 64 (41-81)/65 (28-85)	Dostarlimab + paclitaxel-carboplatin (6 cycles) followed by dostarlimab maintenance (up to 3 years)	Placebo + paclitaxel-carboplatin (6 cycles) followed by placebo maintenance (up to 3 years)	118	376	0	25.4 (range 19.2-37.8)
Pignata et al., 2023 (17)	MITO END-3	Phase 2	31 cancer institutes, hospitals, and universities in Italy	Patients (aged 18 years or older) with histologically confirmed advanced (FIGO stage III-IV) or recurrent endometrial cancer, an ECOG PS of 0-1	63/62	Median (IQR): 66 (61-72)/65 (56-70)	Avelumab + paclitaxel-carboplatin (6-8 cycles) followed by avelumab maintenance (every 3 weeks)	Paclitaxel-carboplatin (6-8 cycles)	57	64	4	23.3 (IQR 13.2-29.6)
Colombo et al., 2024 (24)	AtTEnd	Phase 3	89 hospitals in 11 countries across Europe, Australia, New Zealand, and Asia	Patients (aged 18 years or older) with newly diagnosed EC with measurable or evaluable residual disease after surgery, or inoperable stage III-IV endometrial carcinoma or carcinosarcoma after diagnostic biopsy; ECOG PS of 0-2	360/189	Median (IQR): 67 (61-73)/65 (60-73)	Atezolizumab + paclitaxel-carboplatin (6-8 cycles) followed by atezolizumab maintenance (every 21 days)	Placebo + paclitaxel-carboplatin (6-8 cycles) followed by placebo maintenance (every 21 days)	125	409	15	28.3 (IQR 21.2-37.6)

EC, endometrial cancer; I, intervention group; C, control group; NR, not reported; dMMR, deficient mismatch repair; pMMR, proficient mismatch repair; RECIST, Response Evaluation Criteria for Solid Tumors; ECOG, Eastern Cooperative Oncology Group; PS, performance-status; FIGO, International Federation of Gynecology and Obstetrics; IQR, interquartile range.

### 3.3 Impact of PD-1/PD-L1 inhibitors plus CT on efficacy outcomes

#### 3.3.1 Progression-free survival

**Figure 2:**
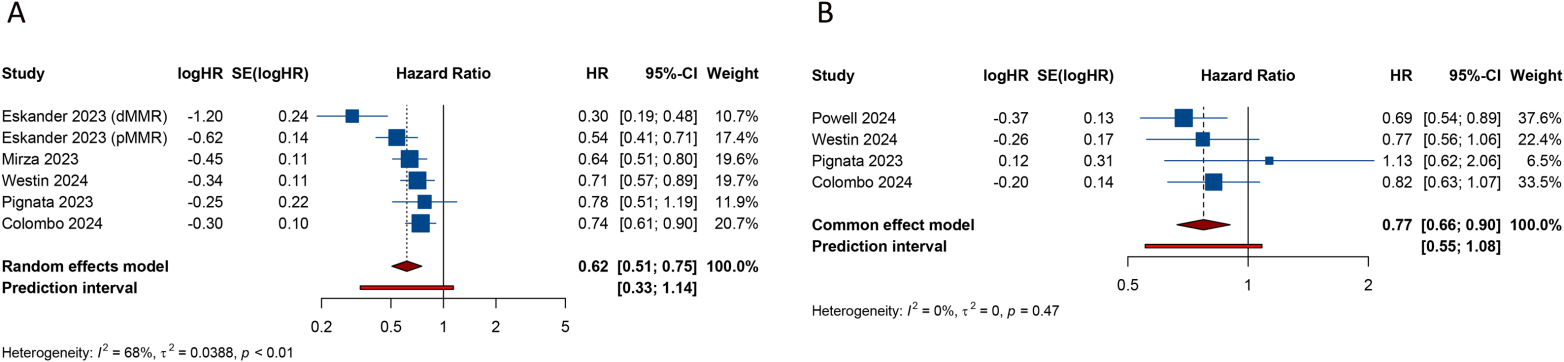
Forest plots of the efficacy outcomes after PD-1/PD-L1 inhibitors combined with chemotherapy for advanced or recurrent endometrial cancer. **(A)** Progression-free survival; **(B)** Overall survival.

All 6 studies evaluated PFS outcome. In patients with advanced or recurrent EC, the estimated PFS rate significantly favored the group receiving PD-1/PD-L1 inhibitors in combination with CT over those receiving CT alone or with a placebo (HR = 0.617, 95% CI: 0.506-0.752; 95% PI: 0.334-1.140, I^2^ = 67.5%) (**Table 2**; **Figure 2A**). Subgroup analyses, based on the types of inhibitors, indicated that both PD-1 (HR = 0.495, 95% CI: 0.346-0.710; 95% PI: 0.008-32.490, I^2^ = 76.0%) and PD-L1 (HR = 0.732, 95% CI: 0.636-0.843; 95% PI: 0.295-1.819, I^2^ = 0%) inhibitors markedly enhanced PFS in EC patients compared to the control group (**Table 2**; **Supplementary Figure 1**). Furthermore, within the dMMR subgroup, the estimated PFS rate notably favored the combination of PD-1/PD-L1 inhibitors and CT (HR = 0.344, 95% CI: 0.269-0.438; 95% PI: 0.231-0.510, I^2^ = 0%) (**Table 2**, **Supplementary Figure 2A**). A similar significant enhancement in PFS was observed with the combination therapy in the pMMR group (HR = 0.772, 95% CI: 0.627-0.950; 95% PI: 0.394-1.512, I^2^ = 62.4%) (**Table 2**; **Supplementary Figure 2B**).

**Table 2:** Pooled effect and subgroup analysis of the efficacy of PD-1/PD-L1 inhibitors combined with chemotherapy in the treatment of advanced or recurrent endometrial cancer.

Outcomes and subgroups	Number of studies	Meta-analysis				Heterogeneity	
		HR	95% CI	*p* value	95% PI	I^2^, Tau^2^	*p* value
PFS							
**Overall population**	6	0.617	0.506-0.752	<0.001	0.334-1.140	67.5%, 0.0388	0.009
PD-1/PD-L1 inhibitors							
PD-1 inhibitors plus CT vs. Placebo plus CT	3	0.495	0.346-0.710	<0.001	0.008-32.490	76.0%, 0.0747	0.016
PD-L1 inhibitors plus CT vs. CT alone (or plus Placebo)	3	0.732	0.636-0.843	<0.001	0.295-1.819	0%, 0	0.919
MMR status							
dMMR	5	0.344	0.269-0.438	<0.001	0.231-0.510	0%, 0	0.762
pMMR	5	0.772	0.627-0.950	0.015	0.394-1.512	62.4%, 0.0344	0.031
Age							
< 65 years	4	0.554	0.387-0.793	0.001	0.126-2.445	67.4%, 0.0856	0.027
≥ 65 years	4	0.567	0.444-0.723	<0.001	0.238-1.351	41.7%, 0.0253	0.161
Race							
White	4	0.556	0.452-0.684	<0.001	0.270-1.143	37.9%, 0.0168	0.185
Asian	3	1.004	0.738-1.365	0.981	0.137-7.358	0%, 0	0.422
Mixed	5	0.620	0.512-0.750	<0.001	0.325-1.089	16.7%, 0.0180	0.308
Histology category							
Endometrioid	4	0.643	0.551-0.750	<0.001	0.459-0.901	0%, 0	0.837
Serous	3	0.710	0.539-0.936	0.015	0.119-4.254	0%, 0	0.562
Mixed	4	0.653	0.516-0.827	<0.001	0.389-1.097	0%, 0	0.633
Disease status							
Newly diagnosed advanced EC	7	0.625	0.459-0.850	0.003	0.253-1.546	62.9%, 0.0995	0.013
Recurrent EC	5	0.636	0.527-0.768	<0.001	0.381-1.063	37.2%, 0.0168	0.174
ECOG performance status							
0	3	0.539	0.345-0.843	0.007	0.003-98.053	76.7%, 0.1156	0.014
1	3	0.489	0.368-0.651	<0.001	0.030-7.539	20.0%, 0.0184	0.286
PD-L1 expression							
Positive	3	0.619	0.495-0.774	<0.001	0.145-2.641	0%, 0	0.857
Negative	3	0.855	0.704-1.039	0.116	0.242-3.022	0%, 0	0.918
Prior CT							
Yes	2	0.680	0.509-0.909	0.009		0%, 0	0.999
No	2	0.647	0.449-0.932	0.019		70.0%, 0.0488	0.068
Prior radiotherapy							
Yes	3	0.588	0.399-0.868	0.007	0.015-23.581	37.7%, 0.0451	0.201
No	3	0.470	0.308-0.717	0.001	0.004-56.645	71.8%, 0.0956	0.029
OS							
**Overall population**	4	0.774	0.664-0.902	0.001	0.553-1.083	0%, 0	0.474
PD-1/PD-L1 inhibitors							
PD-1 inhibitors plus CT vs. Placebo plus CT	1	0.690	0.538-0.886	0.004			
PD-L1 inhibitors plus CT vs. CT alone (or plus Placebo)	3	0.829	0.683-1.006	0.058	0.236-2.916	0%, 0	0.543
MMR status							
dMMR	3	0.371	0.245-0.562	<0.001	0.025-5.461	0%, 0	0.848
pMMR	3	0.996	0.692-1.435	0.983	0.021-47.662	60.6%, 0.0580	0.079

PFS, progression-free survival; CT, chemotherapy; dMMR, deficient mismatch repair; pMMR, proficient mismatch repair; ECOG, Eastern Cooperative Oncology Group; OS, overall survival.

Additionally, we obtained stratified analysis outcomes for PFS based on variables such as age, race, histology category, disease status, Eastern Cooperative Oncology Group (ECOG) performance status, PD-L1 expression, prior CT, and prior radiotherapy from several included studies. These stratified results were synthesized to create subgroup analyses of PFS, as detailed in **Table 2** and **Supplementary Figures 3–10**. Of note, the combination of PD-1/PD-L1 inhibitors with CT showed a significant advantage in enhancing PFS among White patients (HR = 0.556, 95% CI: 0.452-0.684; 95% PI: 0.270-1.143, I^2^ = 37.9%), a benefit not observed in the Asian cohort (HR = 1.004, 95% CI: 0.738-1.365; 95% PI: 0.137-7.358, I^2^ = 0%) (**Table 2**; **Supplementary Figure 4**). Moreover, in EC patients with positive PD-L1 expression, the combination therapy markedly improved PFS (HR = 0.619, 95% CI: 0.495-0.774; 95% PI: 0.145-2.641, I^2^ = 0%), while in those with negative PD-L1 expression, no significant PFS improvement was noted compared to CT alone or plus placebo (HR = 0.855, 95% CI: 0.704-1.039; 95% PI: 0.242-3.022, I^2^ = 0%) (**Table 2**; **Supplementary Figure 8**).

#### 3.3.2 Overall survival

4 studies analyzed the impact of combining PD-1/PD-L1 inhibitors with CT on OS in patients with EC. The pooled estimates revealed a significant enhancement in OS when PD-1/PD-L1 inhibitors were administered alongside CT, compared to CT alone or with a placebo (HR = 0.774, 95% CI: 0.664-0.902; 95% PI: 0.553-1.083, I^2^ = 0%) (**Table 2**; **Figure 2B**). Given the limited availability of studies providing stratified OS analysis, we conducted subgroup analyses based on the types of inhibitors or MMR status. The findings suggested that while PD-L1 inhibitors combined with CT showed a trend toward OS improvement, this did not achieve statistical significance (HR = 0.829, 95% CI: 0.683-1.006; 95% PI: 0.236-2.916, I^2^ = 0%) (**Table 2**; **Supplementary Figure 11A**). In contrast, the addition of PD-1 inhibitors to CT was associated with improved OS (HR = 0.690, 95% CI: 0.538-0.886) (**Table 2**; **Supplementary Figure 11B**), although this finding was derived from a single study. Moreover, the combination of PD-1/PD-L1 inhibitors with CT significantly enhanced OS in patients with dMMR (HR = 0.371, 95% CI: 0.245-0.562; 95% PI: 0.025-5.461, I^2^ = 0%), but not in those with pMMR (HR = 0.996, 95% CI: 0.692-1.435; 95% PI: 0.021-47.662, I^2^ = 60.6%) (**Table 2**; **Supplementary Figure 12**).

### 3.4 Impact of PD-1/PD-L1 inhibitors plus CT on safety outcomes

#### 3.4.1 Any grade adverse events

**Figure 3:**
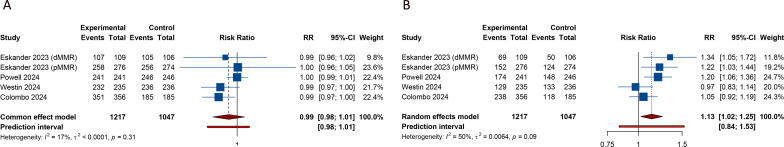
Forest plots of the safety outcomes after PD-1/PD-L1 inhibitors combined with chemotherapy for advanced or recurrent endometrial cancer. **(A)** Any grade adverse events; **(B)** Grade ≥ 3 adverse events.

5 studies assessed the incidence of AEs of any grade in the experimental and control groups. The comprehensive analysis revealed no significant difference in the risk of any grade AEs between the group receiving PD-1/PD-L1 inhibitors with CT and the group receiving CT with a placebo (RR = 0.994, 95% CI: 0.982-1.006; 95% PI: 0.978-1.009, I^2^ = 16.7%) (**Table 3**; **Figure 3A**). The common AEs of any grade, as identified from the included RCTs, encompassed blood and lymphatic system disorders (e.g., anemia, thrombocytopenia, and neutropenia), gastrointestinal disorders (nausea, constipation, diarrhea, and vomiting), musculoskeletal and connective tissue disorders (arthralgia and myalgia), skin and subcutaneous tissue disorders (alopecia and rash), and other symptoms (fatigue, peripheral sensory neuropathy, dyspnea, decreased appetite, and urinary tract infection). Compared with CT alone (or plus placebo), the combination of PD-1/PD-L1 inhibitors with CT significantly elevated the risk of thrombocytopenia (RR = 1.226, 95% CI: 1.048-1.434; 95% PI: 0.867-1.727, I^2^ = 0%) and vomiting (RR = 1.471, 95% CI: 1.179-1.835; 95% PI: 0.891-2.392, I^2^ = 11.1%), while decreasing the likelihood of urinary tract infection (RR = 0.698, 95% CI: 0.516-0.943; 95% PI: 0.099-4.930, I^2^ = 0%) (**Table 3**). No significant differences were observed in the occurrence of anemia, neutropenia, nausea, constipation, diarrhea, arthralgia, myalgia, alopecia, rash, fatigue, peripheral sensory neuropathy, dyspnea, and decreased appetite between the experimental and control group (all *p* > 0.05) (**Table 3**; **Supplementary Figures 13-17**).

**Table 3:** Pooled effect of the safety of PD-1/PD-L1 inhibitors combined with chemotherapy in the treatment of advanced or recurrent endometrial cancer.

Outcomes and Events	Number of studies	Meta-analysis				Heterogeneity	
		RR	95% CI	*p* value	95% PI	I^2^, Tau^2^	*p* value
**Any grade AEs**	5	0.994	0.982-1.006	0.300	0.978-1.009	16.7%, <0.0001	0.308
Blood and lymphatic system disorders							
Anemia	6	0.995	0.914-1.084	0.913	0.857-1.156	7.0%, 0.0009	0.372
Thrombocytopenia	4	1.226	1.048-1.434	0.011	0.867-1.727	0%, 0	0.579
Neutropenia	4	0.978	0.835-1.146	0.786	0.586-1.633	29.5%, 0.0077	0.236
Gastrointestinal disorders							
Nausea	6	1.039	0.946-1.140	0.425	0.872-1.252	10.5%, 0.0017	0.348
Constipation	6	1.011	0.903-1.131	0.851	0.825-1.250	8.4%, 0.0019	0.363
Diarrhea	6	1.128	0.994-1.279	0.061	0.945-1.349	0%, 0	0.994
Vomiting	5	1.471	1.179-1.835	0.001	0.891-2.392	11.1%, 0.0089	0.343
Musculoskeletal and connective tissue disorders							
Arthralgia	6	1.006	0.884-1.144	0.934	0.840-1.211	0%, 0	0.562
Myalgia	4	1.123	0.877-1.438	0.358	0.496-2.545	31.6%, 0.0202	0.223
Skin and subcutaneous tissue disorders							
Alopecia	4	0.958	0.820-1.119	0.588	0.548-1.676	43.7%, 0.0106	0.149
Rash	4	1.468	0.885-2.437	0.138	0.200-10.777	61.7%, 0.1478	0.049
Other							
Fatigue	5	1.069	0.951-1.201	0.262	0.767-1.490	43.4%, 0.0074	0.133
Peripheral sensory neuropathy	6	0.990	0.898-1.091	0.839	0.867-1.132	0%, 0	0.935
Dyspnea	4	1.080	0.868-1.343	0.493	0.667-1.747	0%, 0	0.516
Decreased appetite	3	1.033	0.821-1.301	0.782	0.232-4.625	0%, 0	0.468
Urinary tract infection	3	0.698	0.516-0.943	0.019	0.099-4.930	0%, 0	0.876
**Grade ≥ 3 AEs**	5	1.132	1.023-1.252	0.016	0.836-1.532	49.7%, 0.0064	0.093
Blood and lymphatic system disorders							
Anemia	6	1.177	0.960-1.442	0.117	0.870-1.569	0.4%, 0.0003	0.413
Thrombocytopenia	4	1.390	0.928-2.081	0.110	0.565-3.360	0%, 0	0.760
Neutropenia	5	1.035	0.837-1.281	0.749	0.611-1.753	26.5%, 0.0156	0.245
Gastrointestinal disorders							
Nausea	4	1.265	0.492-3.249	0.626	0.148-10.208	0%, 0	0.873
Constipation	4	0.992	0.272-3.614	0.991	0.0002-5763.698	0%, 0	0.727
Diarrhea	4	1.795	0.419-7.684	0.431	0.010-313.724	40.7%, 0.8898	0.168
Vomiting	4	1.320	0.463-3.765	0.604	0.121-13.058	0%, 0	0.896
Other							
Fatigue	4	0.968	0.486-1.928	0.926	0.199-4.672	0%, 0	0.487
Peripheral sensory neuropathy	4	1.179	0.581-2.395	0.648	0.136-8.121	6.9%, 0.0552	0.359
Arthralgia	4	0.740	0.255-2.153	0.581	0.001-999.142	0%, 0	0.595
Hypertension	3	1.953	1.134-3.366	0.016	0.053-69.830	0%, 0	0.414

AEs, adverse events.

#### 3.4.2 Grade ≥ 3 adverse events

Data from 5 studies indicated a significantly higher incidence of grade ≥ 3 AEs in patients treated with a combination of PD-1/PD-L1 inhibitors and CT compared to those receiving CT plus placebo (RR = 1.132, 95% CI: 1.023-1.252; 95% PI: 0.836-1.532, I^2^ = 49.7%) (**Table 3**; **Figure 3B**). The frequent grade ≥ 3 AEs included blood and lymphatic system disorders (anemia, thrombocytopenia, and neutropenia), gastrointestinal disorders (nausea, constipation, diarrhea, and vomiting), as well as other conditions such as fatigue, peripheral sensory neuropathy, arthralgia, and hypertension. Notably, the combination of PD-1/PD-L1 inhibitors with CT was associated with an increased risk of hypertension relative to the control group (RR = 1.953, 95% CI: 1.134-3.366; 95% PI: 0.053-69.830, I^2^ = 0%) (**Table 3**). However, this combination therapy did not elevate the risks of anemia, thrombocytopenia, neutropenia, nausea, constipation, diarrhea, vomiting, fatigue, peripheral sensory neuropathy, or arthralgia when compared to CT alone or with placebo (all *p* > 0.05) (**Table 3**; **Supplementary Figures 18-20**).

### 3.5 Trial sequential analysis results

**Figure 4:**
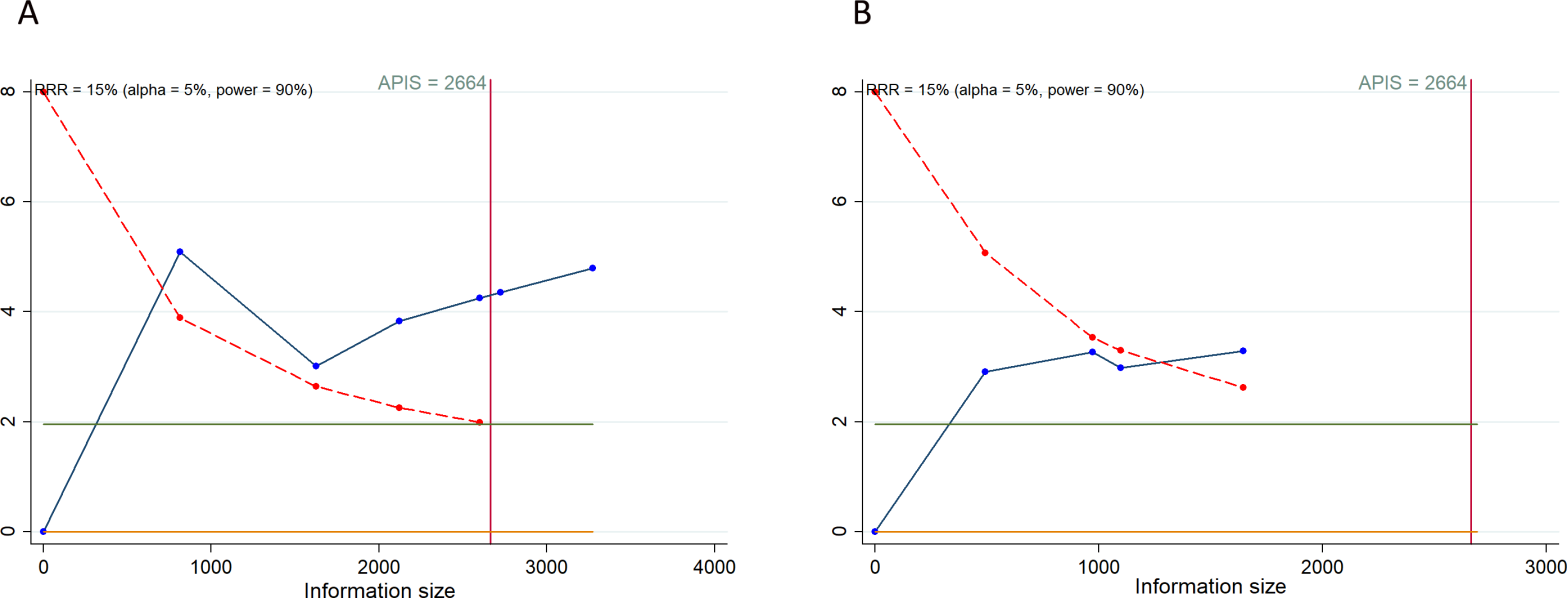
Trial sequential analysis of PD-1/PD-L1 inhibitors combined with chemotherapy for advanced or recurrent endometrial cancer. **(A)** Progression-free survival; **(B)** Overall survival. Red inward-sloping line to the left represents trial sequential monitoring boundary. Blue line represents evolution of cumulative Z-score. Horizontal green lines represent the conventional boundaries for statistical significance. Heterogeneity-adjusted required information size to demonstrate or reject 15% relative risk (*a priori* estimate) of mortality risk (with alpha of 5% and beta of 10%) is 2664 patients for PFS and OS (vertical red line). Cumulative Z-curve crossing the trial sequential monitoring boundary or the APIS boundary provides firm evidence of effect.

**Figure 5:**
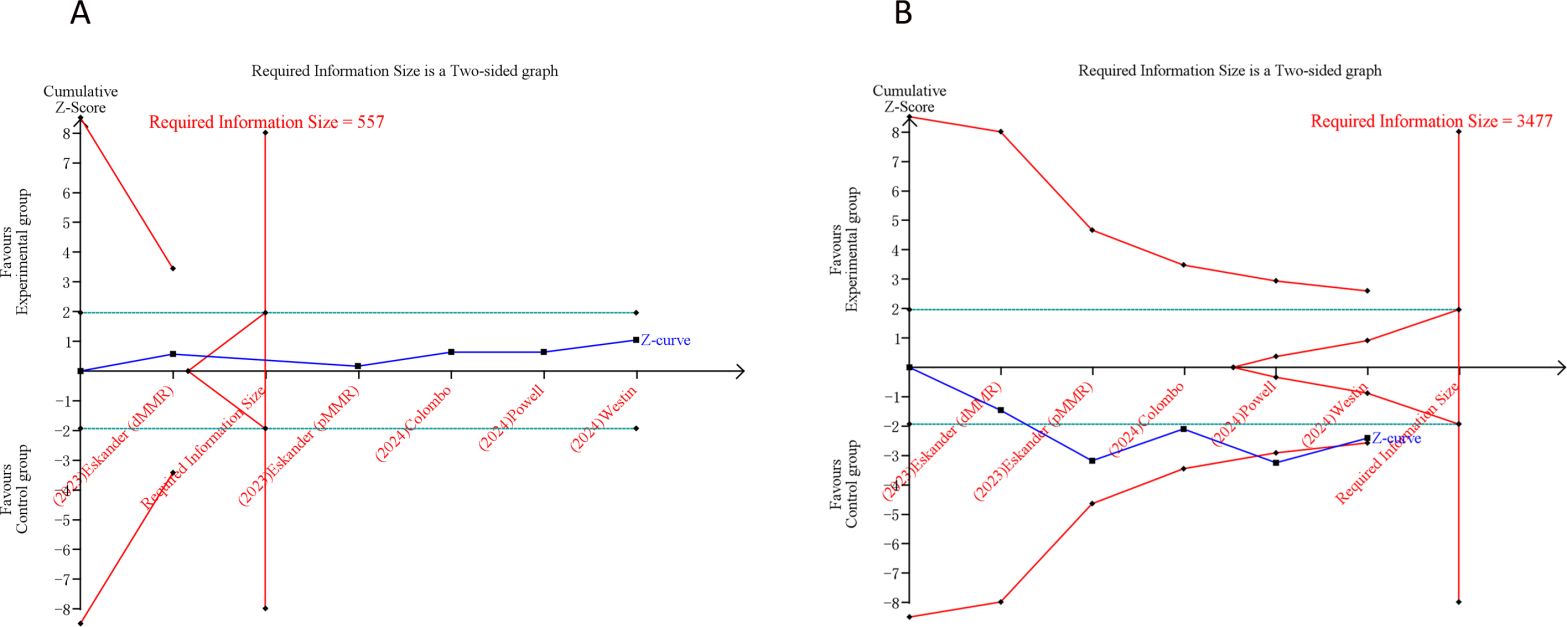
Trial sequential analysis of PD-1/PD-L1 inhibitors combined with chemotherapy for advanced or recurrent endometrial cancer. **(A)** Any grade adverse events; **(B)** Grade ≥ 3 adverse events. Uppermost and lowermost red curves represent trial sequential monitoring boundary lines for benefit and harm, respectively. Inner red lines represent the futility boundary. Blue line represents evolution of cumulative Z-score. Horizontal green lines represent the conventional boundaries for statistical significance. Cumulative Z-curve crossing the trial sequential monitoring boundary or the RIS boundary provides firm evidence of effect.

In our TSA for PFS and OS, we established an APIS of 2,664. The TSA for PFS revealed that the cumulative Z-curve surpassed both the APIS and the trial sequential monitoring boundaries (**Figure 4A**). For OS, the cumulative Z-curve crossed the trial sequential monitoring boundary but did not exceed the APIS (**Figure 4B**). Consequently, no further testing is necessary, and the findings for PFS and OS are reliable and conclusive. Similarly, the cumulative Z-curve for any grade AEs exceeded both the RIS and the trial sequential monitoring boundaries, while for grade ≥ 3 AEs, it crossed the trial sequential monitoring boundary without surpassing the RIS boundary (**Figure 5**). This provides robust evidence for the impact of PD-1/PD-L1 inhibitors in combination with CT on any grade and grade ≥ 3 AEs compared to the control group.

### 3.6 Sensitivity analysis and publication bias

We conducted a leave-one-out sensitivity analysis to evaluate the impact of each individual study on the overall pooled HRs for PFS and OS, as well as the pooled RRs for AEs of any grade and those of grade ≥ 3. Due to the small number of studies included, the sensitivity analysis revealed that excluding the study by Powell et al. impacted the overall findings for OS and grade ≥ 3 AEs. However, no single study significantly altered the results for PFS or any grade AEs, demonstrating the stability of these findings (**Supplementary Figure 21**). To further assess publication bias, we employed a combination of funnel plots alongside Begg’s and Egger’s tests, both of which indicated no evidence of publication bias in the efficacy and safety outcomes (all *p* > 0.05). The corresponding funnel plots were provided in **Supplementary Figure 22**.

## 4 Discussion

Advanced or recurrent EC is associated with a dismal prognosis and a recurrence rate ranging from 40% to 70% (38). This malignancy significantly affects women’s health, contributing to high levels of morbidity and mortality, particularly in patients who do not respond to platinum-based therapies (39, 40). Therefore, identifying effective treatments beyond first-line options remains a critical unmet need (40). Recently, immunotherapy has emerged as a promising approach for advanced or recurrent EC, with a particular focus on ICIs targeting PD-1 and PD-L1 (41, 42). Numerous investigations have been carried out to enhance and substantiate the efficacy of these novel ICIs across a range of cancers, including EC. This meta-analysis pooled data on the efficacy and safety of PD-1/PD-L1 inhibitors combined with CT versus CT alone (or plus placebo) in patients with advanced or recurrent EC. The main findings indicated that the combination of PD-1/PD-L1 inhibitors with CT improved PFS irrespective of MMR status. While the combination therapy also significantly enhanced OS compared with CT alone or with placebo in the overall population, this benefit was confined to patients with dMMR and was not significant in those with pMMR. The results of the TSA analysis indicated that the findings for PFS and OS are robust and conclusive.

The improvements in PFS and OS observed with PD-1/PD-L1 inhibitors in patients with advanced or recurrent EC can be attributed to specific biological mechanisms. These include the modulation of molecular pathways and immunological interactions mediated by these therapies, as well as their synergistic effects when combined with CT. PD-1 is a receptor predominantly expressed on T cells and is present in approximately 90% of EC cases (43). PD-L1 interacts with PD-1, leading to the phosphorylation of PD-1 by the protein tyrosine kinase Lck. This process subsequently recruits Src homology region 2 domain-containing phosphatase-2 (SHP2), which dephosphorylates the T-cell receptor (TCR) and CD28, ultimately inhibiting T-cell signaling and function. The introduction of PD-1/PD-L1 inhibitors disrupts this phosphorylation cascade, preventing SHP2 recruitment and allowing for sustained activation of TCR and CD28, thereby facilitating T-cell proliferation and differentiation (44–47). Importantly, PD-1/PD-L1 inhibitors do not directly kill cancer cells; instead, they block the interaction between PD-1 and PD-L1, disrupting the inhibitory signaling mediated by these molecules. This blockade activates T cells, thereby enhancing the patient’s immune defense mechanisms and exerting an anti-tumor effect (48). Moreover, the therapeutic potential of combining PD-1/PD-L1 inhibitors with CT for patients with advanced or recurrent EC is supported by several mechanisms. Notably, genetic mutations arising from clonal evolution increase tumor antigenic diversity, which may interact synergistically with the immunogenic effects of CT. This interaction can elevate the ratio of cytotoxic T lymphocytes to regulatory T cells (T(regs)). Furthermore, this combinatorial therapy has the potential to boost the activation of dendritic cells (DCs) by targeting the STAT6 pathway. It also promotes effective antigen cross-presentation and suppresses myeloid-derived suppressor cells. Together, these mechanisms establish an environment that is favorable for a positive therapeutic response (49–52).

Our subgroup analysis has revealed that combining PD-1/PD-L1 inhibitors with CT yielded superior PFS and OS benefits in EC patients with dMMR. While patients with pMMR also experienced a PFS advantage, it is notably less pronounced compared to those with dMMR (pMMR: HR = 0.772 vs. dMMR: HR = 0.344). Individuals with advanced or recurrent EC who are categorized as having dMMR could exhibit greater responsiveness to PD-1/PD-L1 inhibitors. This increased sensitivity is likely due to the elevated levels of PD-1 and PD-L1 expressed within their tumor microenvironment (TME) compared to those with pMMR (53). Subsequent research has demonstrated that the ORR is 46% in dMMR patients with advanced or recurrent EC, compared to 13% in their pMMR counterparts following treatment with PD-1/PD-L1 inhibitors (54). The recent AtTEnd trial revealed that the addition of the PD-L1 inhibitor atezolizumab to standard first-line CT markedly enhanced PFS in patients with advanced or recurrent EC across both the dMMR subset and the overall cohort. However, this improvement was not observed in the pMMR subgroup. The overall PFS benefit from atezolizumab was primarily attributable to its effect in the dMMR population (24).

Additionally, our subgroup analyses based on PD-L1 expression indicated a PFS advantage in the PD-L1 positive cohort, whereas no significant benefit was observed in the PD-L1 negative group. The utility of PD-L1 as a biomarker remains complex due to its variable expression, particularly its propensity to upregulate in response to immunotherapy (55, 56). The KEYNOTE-018 trial, a phase Ib investigation into the safety and effectiveness of pembrolizumab in EC, found that PD-L1 expression assessed through immunohistochemistry served as a limited prognostic indicator. Notably, some patients who were PD-L1 positive did not respond to pembrolizumab as anticipated (15). Thus, additional studies are necessary to ascertain the most effective role and use of this biomarker.

Interestingly, our subgroup analysis revealed that White patients experienced a significant PFS benefit from the combination of PD-1/PD-L1 inhibitors and CT, whereas Asian patients did not show a notable PFS improvement. Several hypotheses might explain this discrepancy. Firstly, the impact of PD-1/PD-L1 inhibitors on prognosis is influenced by racial variations in molecular aberrations. A national cohort study conducted in Japan found a greater incidence of POLE mutations (18%), along with dMMR (27%) and p53 abnormalities (28%), compared to research involving more diverse populations (57). Additionally, an analysis of The Cancer Genome Atlas Endometrial Cancer dataset indicated that Asian individuals displayed elevated rates of somatic mutations in MMR genes such as MSH2, MSH6, MLH1, and PMS2 when compared to Caucasian individuals (58). Moreover, the majority of participants in the included RCTs were European, with only a small proportion being Asian, leading to a wide 95% CI for the HR of PFS in the Asian subgroup, which may result in non-significant findings. Therefore, to gain a deeper insight into the molecular disparities linked to race, it would be imperative to undertake large-scale, multinational studies. Of note, subgroup analysis according to the types of inhibitors demonstrated that the combination of PD-1 or PD-L1 inhibitors with CT improved PFS. Additionally, our meta-analysis revealed that the combination of PD-1/PD-L1 inhibitors with CT significantly improved PFS, regardless of patient age (< 65 years or ≥ 65 years), histology category (endometrioid, serous or mixed), disease status (newly diagnosed advanced EC or recurrent EC), ECOG performance status (0 or 1), prior CT history (yes or no) or radiotherapy history (yes or no). This observation, which has not been reported in previous studies, further supports the consistency of PD-1/PD-L1 inhibitors in improving PFS among patients with advanced or recurrent EC.

Safety is a crucial element in all innovative research endeavors. The RCTs analyzed in this meta-analysis documented AEs associated with treatments. Common AEs of any grade reported in both experimental and control groups included anemia, thrombocytopenia, neutropenia, nausea, constipation, diarrhea, vomiting, arthralgia, myalgia, alopecia, rash, fatigue, peripheral sensory neuropathy, dyspnea, decreased appetite, and urinary tract infection. Notably, the combination of PD-1/PD-L1 inhibitors with CT was associated with an elevated risk of any grade thrombocytopenia and vomiting, while it mitigated the risk of urinary tract infection relative to CT alone or plus placebo. For grade ≥ 3 AEs, frequent occurrences were anemia, thrombocytopenia, neutropenia, nausea, constipation, diarrhea, vomiting, fatigue, peripheral sensory neuropathy, arthralgia, and hypertension. The only notable difference between the experimental and control cohorts was in the incidence of hypertension, with combination therapy presenting a greater risk. These AEs may be gradually ameliorated through dose reduction or cessation of the drug (59). Presently, while minor variations exist in AEs across different PD-1/PD-L1 inhibitors combined with CT, the overall efficacy is significant and toxicity remains manageable compared to CT alone. To ensure the appropriate management of AEs, it is imperative that the safety of this combination therapy are rigorously monitored and evaluated in ongoing clinical trials (60).

This study is subject to several limitations. First, this meta-analysis was based on studies without integrating individual patient data, introducing an unavoidable degree of selection bias. Second, moderate heterogeneity was observed in the pooled PFS analysis. This heterogeneity may stem from differences in MMR status among EC patients, as well as differences in race, histology category, and PD-L1 expression, as indicated by subgroup analyses. Third, the inclusion of 5 trials utilizing various PD-1/PD-L1 inhibitors-such as pembrolizumab, dostarlimab, durvalumab, avelumab, and atezolizumab-necessitates further RCTs to comprehensively assess the efficacy and safety of these agents in EC patients. Fourth, the limited number of RCTs included resulted in insufficient mature data on the impact of combining PD-1/PD-L1 inhibitors with CT on OS. Therefore, caution is warranted in interpreting these findings, and additional forthcoming data are highly anticipated.

## 5 Conclusion

In summary, the combination of PD-1/PD-L1 inhibitors with CT has been demonstrated to significantly improve PFS and OS for patients with advanced or recurrent EC. Notably, patients characterized by dMMR status, White ethnicity, or positive PD-L1 expression may exhibit pronounced benefits from this therapeutic strategy. However, this treatment regimen also led to a marked increase in the occurrence of grade ≥ 3 AEs. These findings suggest that tailoring treatment based on specific patient characteristics could optimize outcomes, and it is crucial for clinicians to remain vigilant for potential AEs.

## Data Availability

The original contributions presented in the study are included in the article/**Supplementary Material**. Further inquiries can be directed to the corresponding author.
