# Supplementary material for: Efficacy and safety of PD-1/PD-L1 inhibitors in advanced or recurrent endometrial cancer: a meta-analysis with trial sequential analysis of randomized controlled trials
Source: Front Immunol. 2025 Jan 31;16:1521362. doi: 10.3389/fimmu.2025.1521362 (PMC11825832; doi:10.3389/fimmu.2025.1521362)
Supplement: Supplementary file 1 [file DataSheet1.docx]

| **PubMed 953** |
| --- |
| #1 (immune checkpoint inhibitor) OR (immune checkpoint inhibitors) OR (ICI) OR (PD-1) OR (PD-L1) OR (programmed cell death protein 1 inhibitor) OR (programmed death-ligand 1 inhibitor) OR (pembrolizumab) OR (atezolizumab) OR (dostarlimab) OR (durvalumab) OR (nivolumab) OR (avelumab) OR (keytruda) |
| #2 (endometrial cancer) OR (endometrium cancer) OR (cancer of endometrium) OR (endometrial neoplasms) OR (endometrial carcinoma) OR (endometrium carcinoma) |
| #3 #1 AND #2 |
| **Web of Science 1389** |
| #1 TS=((immune checkpoint inhibitor) OR (immune checkpoint inhibitors) OR (ICI) OR (PD-1) OR (PD-L1) OR (programmed cell death protein 1 inhibitor) OR (programmed death-ligand 1 inhibitor) OR (pembrolizumab) OR (atezolizumab) OR (dostarlimab) OR (durvalumab) OR (nivolumab) OR (avelumab) OR (keytruda)) |
| #2 TS=((endometrial cancer) OR (endometrium cancer) OR (cancer of endometrium) OR (endometrial neoplasms) OR (endometrial carcinoma) OR (endometrium carcinoma)) |
| #3 #1 AND #2 |
| **The Cochrane Library 212** |
| #1 All Text=((immune checkpoint inhibitor) OR (immune checkpoint inhibitors) OR (ICI) OR (PD-1) OR (PD-L1) OR (programmed cell death protein 1 inhibitor) OR (programmed death-ligand 1 inhibitor) OR (pembrolizumab) OR (atezolizumab) OR (dostarlimab) OR (durvalumab) OR (nivolumab) OR (avelumab) OR (keytruda)) |
| #2 All Text=((endometrial cancer) OR (endometrium cancer) OR (cancer of endometrium) OR (endometrial neoplasms) OR (endometrial carcinoma) OR (endometrium carcinoma)) |
| #3 #1 AND #2 |
| **Embase 1380** |
| #1 'immune checkpoint inhibitor':ti,ab,kw OR 'immune checkpoint inhibitors':ti,ab,kw OR ici:ti,ab,kw OR 'pd 1':ti,ab,kw OR 'pd l1':ti,ab,kw OR 'programmed cell death protein 1 inhibitor':ti,ab,kw OR 'programmed death-ligand 1 inhibitor':ti,ab,kw OR pembrolizumab:ti,ab,kw OR atezolizumab:ti,ab,kw OR dostarlimab:ti,ab,kw OR durvalumab:ti,ab,kw OR nivolumab:ti,ab,kw OR avelumab:ti,ab,kw OR keytruda:ti,ab,kw |
| #2 'endometrial cancer':ti,ab,kw OR 'endometrium cancer':ti,ab,kw OR 'cancer of endometrium':ti,ab,kw OR 'endometrial neoplasms':ti,ab,kw OR 'endometrial carcinoma':ti,ab,kw OR 'endometrium carcinoma':ti,ab,kw |
| #3 #1 AND #2 |
