# Supplementary material for: Efficacy and safety of PD-1/PD-L1 inhibitors in advanced or recurrent endometrial cancer: a meta-analysis with trial sequential analysis of randomized controlled trials
Source: Front Immunol. 2025 Jan 31;16:1521362. doi: 10.3389/fimmu.2025.1521362 (PMC11825832; doi:10.3389/fimmu.2025.1521362)

**FIGURE S1** Subgroup analysis of progression-free survival based on the types of inhibitors. (A) PD-1 inhibitors; (B) PD-L1 inhibitors.


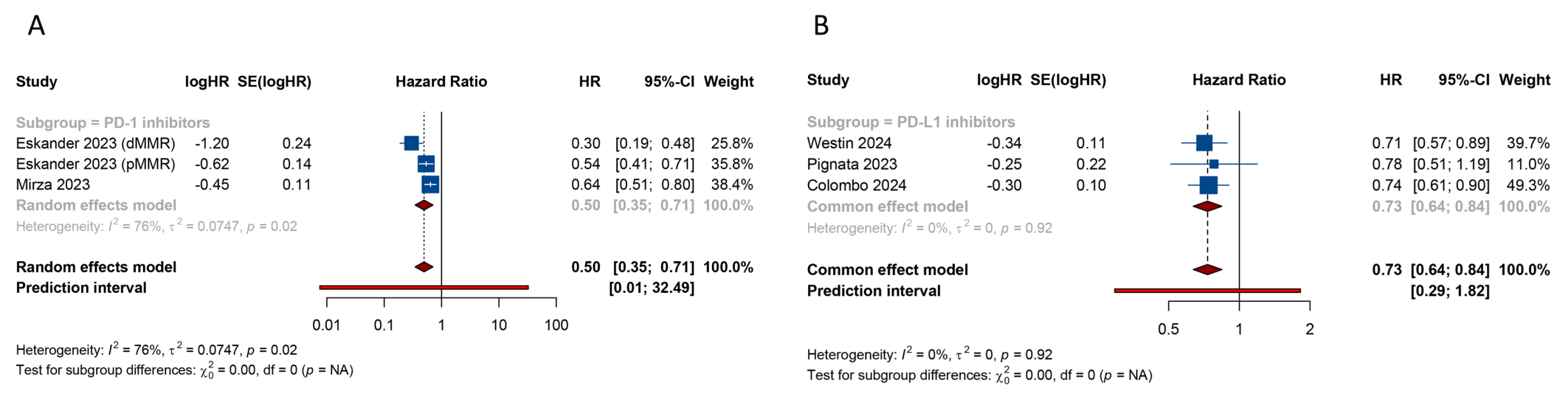


**FIGURE S2** Subgroup analysis of progression-free survival based on mismatch repair (MMR) status. (A) Deficient mismatch repair (dMMR); (B) Proficient mismatch repair (pMMR).


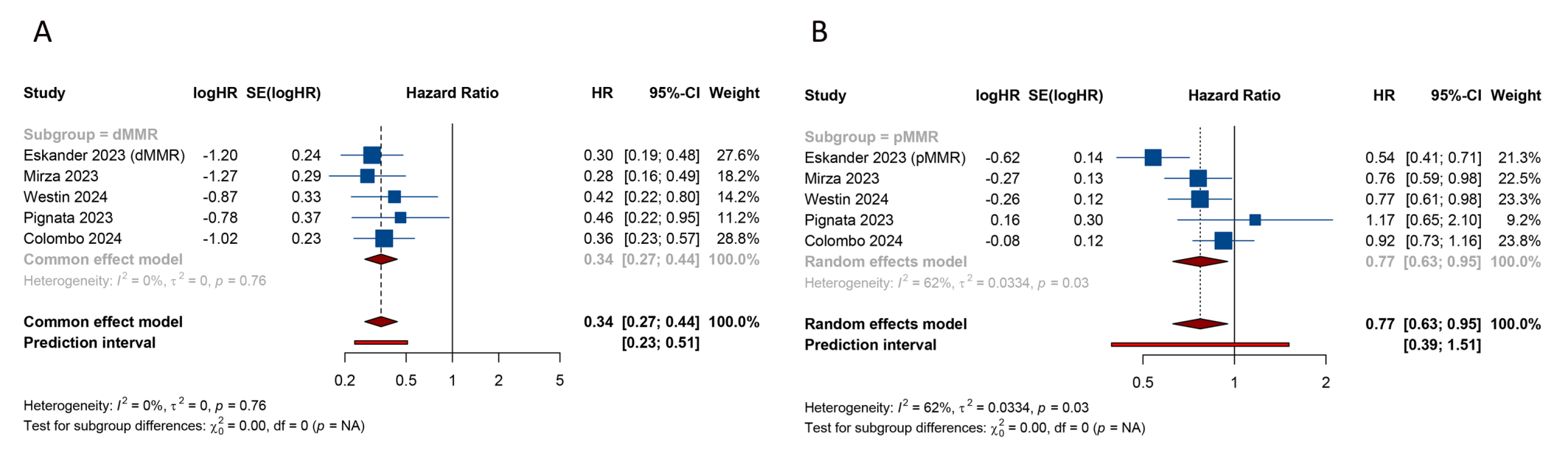


**FIGURE S3** Subgroup analysis of progression-free survival based on the age of participants. (A) < 65 years; (B) ≥ 65 years.


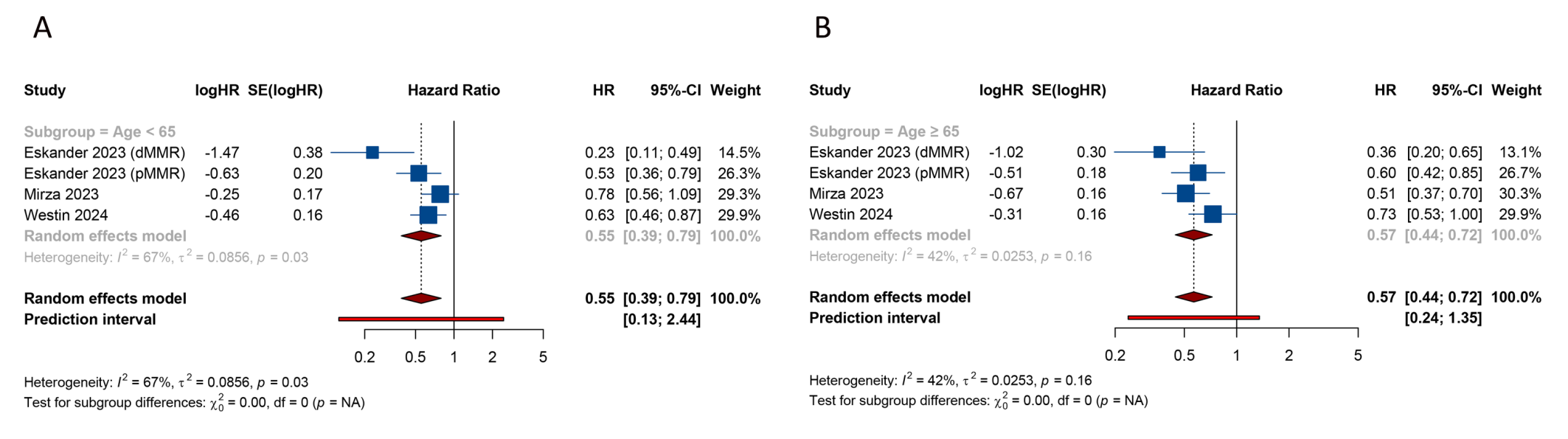


**FIGURE S4** Subgroup analysis of progression-free survival based on participant race. (A) White; (B) Asian; (C) Mixed.


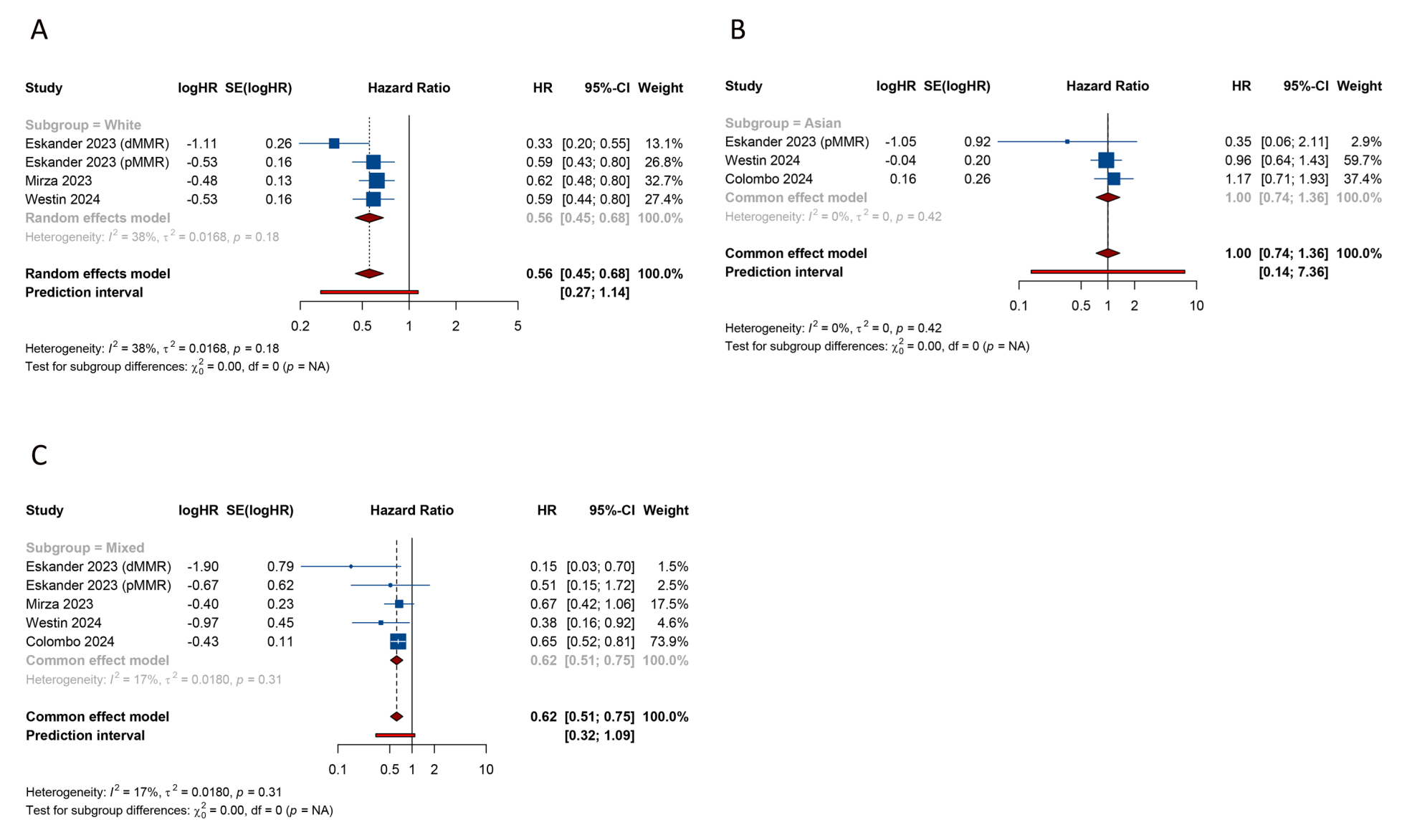


**FIGURE S5** Subgroup analysis of progression-free survival based on histology category. (A) Endometrioid; (B) Serous; (C) Mixed.


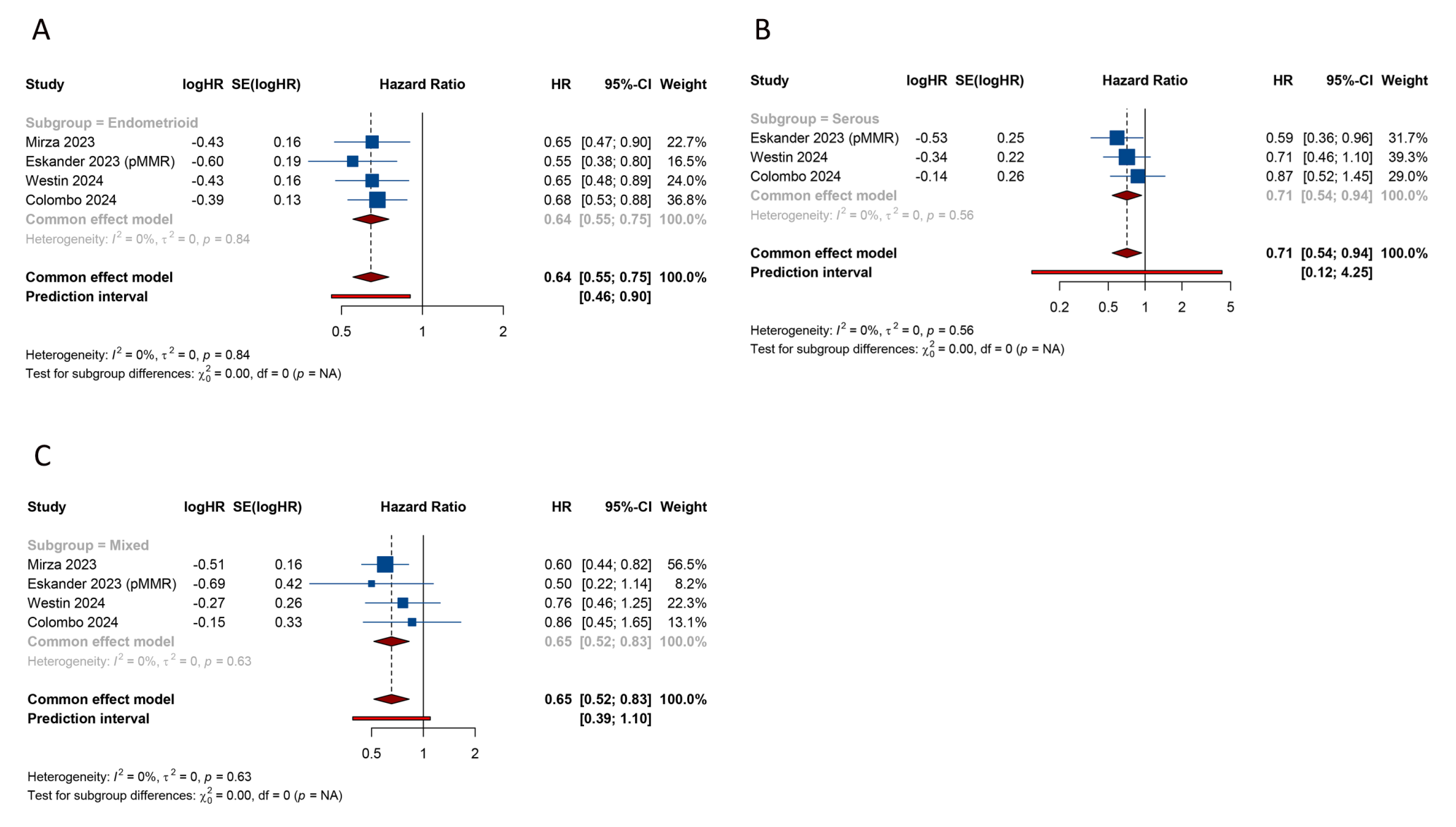


**FIGURE S6** Subgroup analysis of progression-free survival based on disease status. (A) Newly diagnosed advanced endometrial cancer (EC); (B) Recurrent EC.


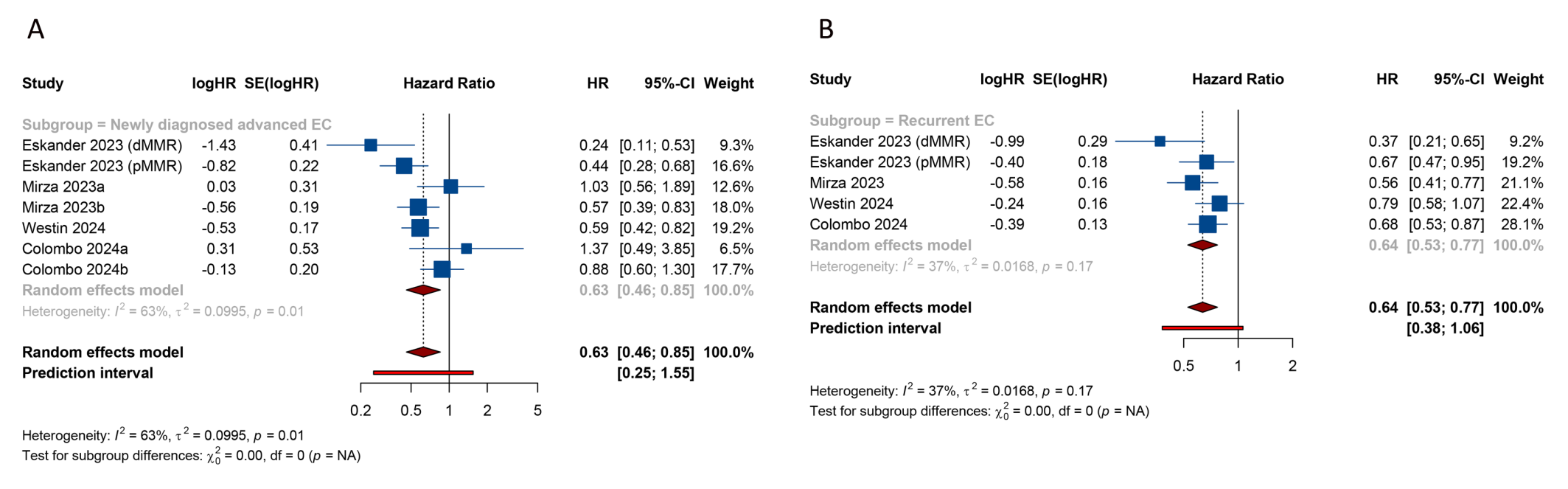


**FIGURE S7** Subgroup analysis of progression-free survival based on Eastern Cooperative Oncology Group (ECOG) performance status (PS). (A) ECOG PS of 0; (B) ECOG PS of 1.


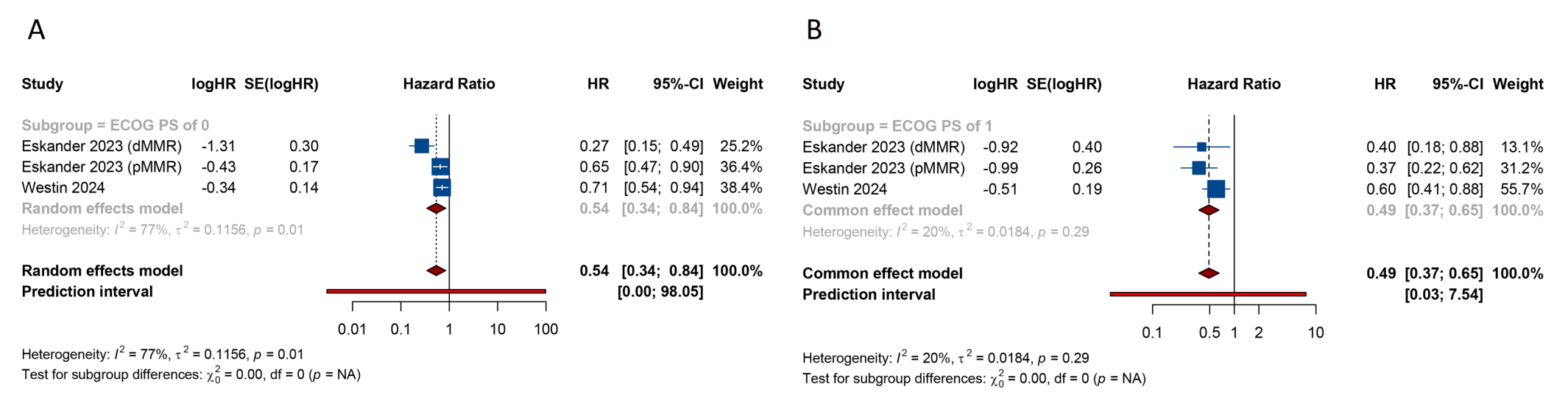


**FIGURE S8** Subgroup analysis of progression-free survival based on PD-L1 expression. (A) PD-L1 expression positive; (B) PD-L1 expression negative.


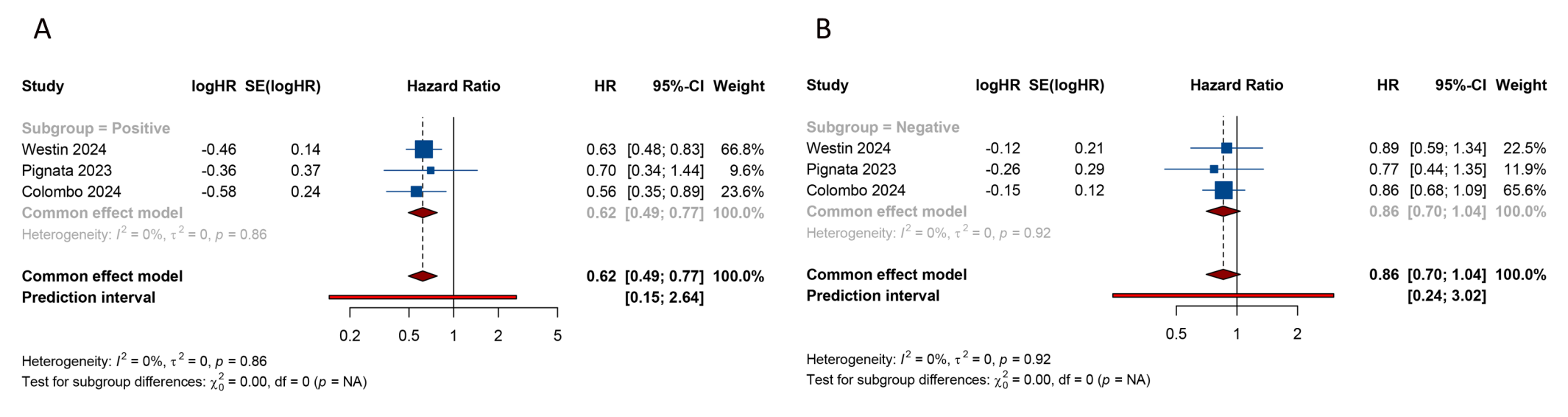


**FIGURE S9** Subgroup analysis of progression-free survival based on prior chemotherapy. (A) Prior chemotherapy = Yes; (B) Prior chemotherapy = No.


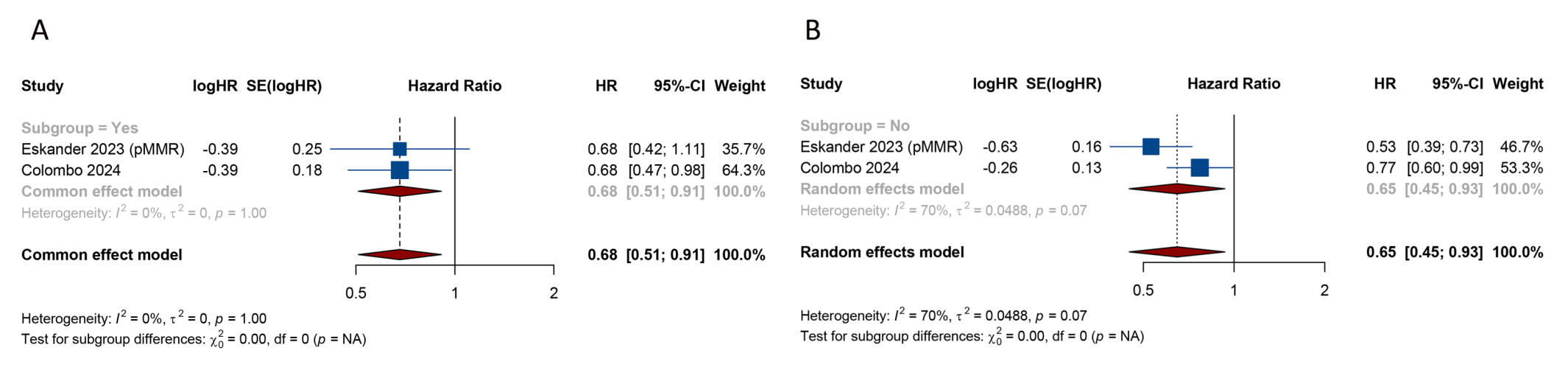


**FIGURE S10** Subgroup analysis of progression-free survival based on prior radiotherapy. (A) Prior radiotherapy = Yes; (B) Prior radiotherapy = No.


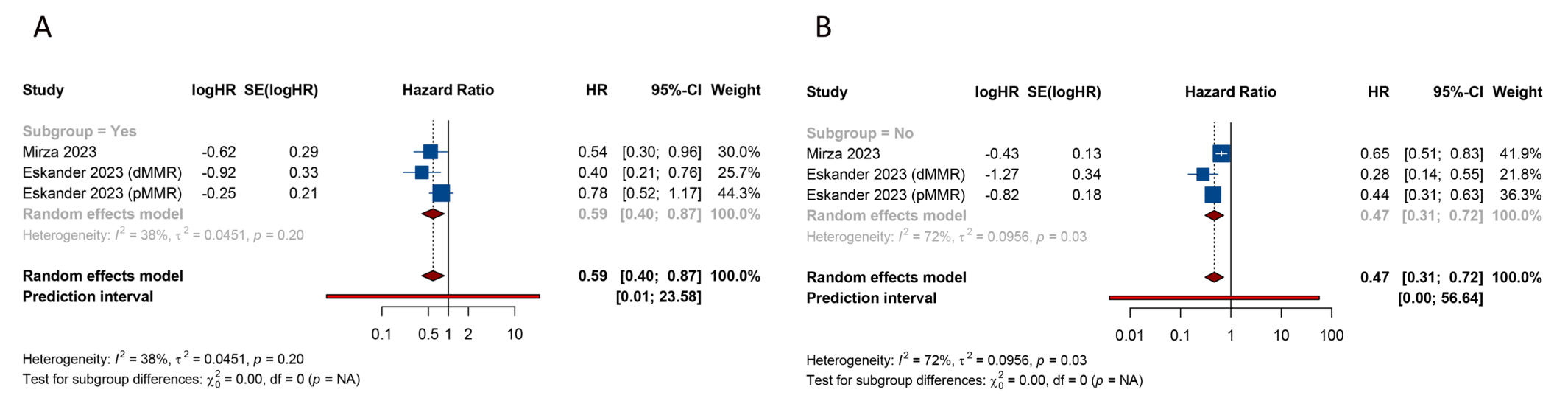


**FIGURE S11** Subgroup analysis of overall survival based on the types of inhibitors. (A) PD-L1 inhibitors; (B) PD-1 inhibitors.


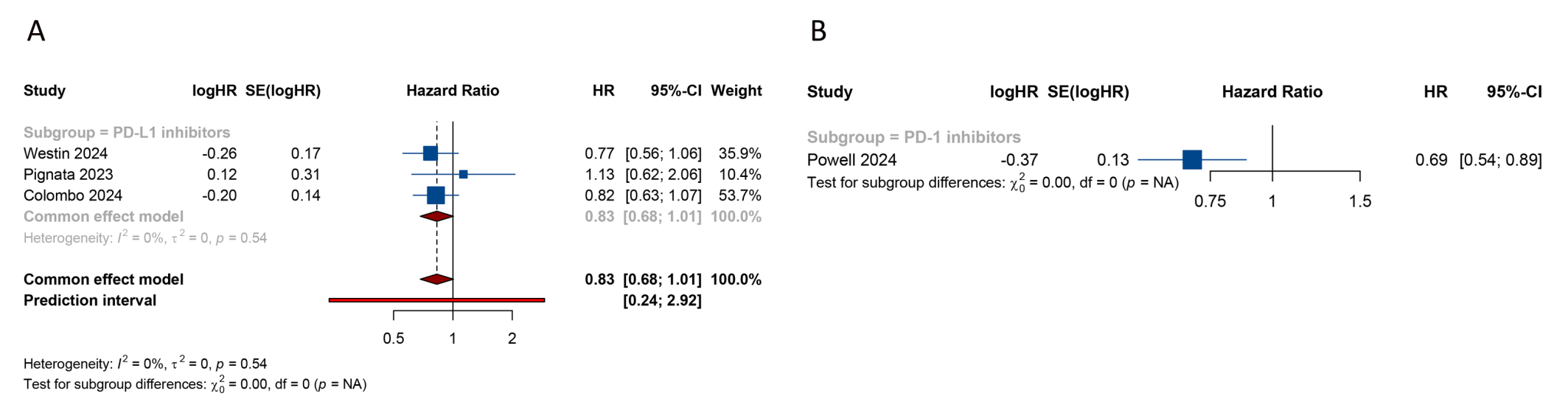


**FIGURE S12** Subgroup analysis of overall survival based on mismatch repair (MMR) status. (A) Deficient mismatch repair (dMMR); (B) Proficient mismatch repair (pMMR).


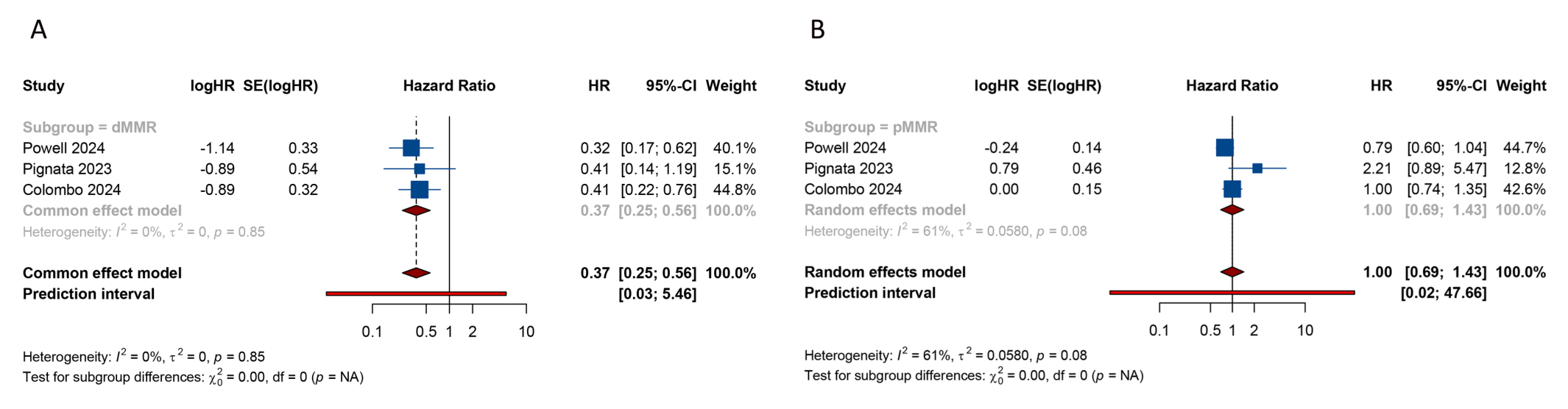


**FIGURE S13** Forest plots of any grade adverse events of blood and lymphatic system disorders. (A) Anemia; (B) Thrombocytopenia; (C) Neutropenia.


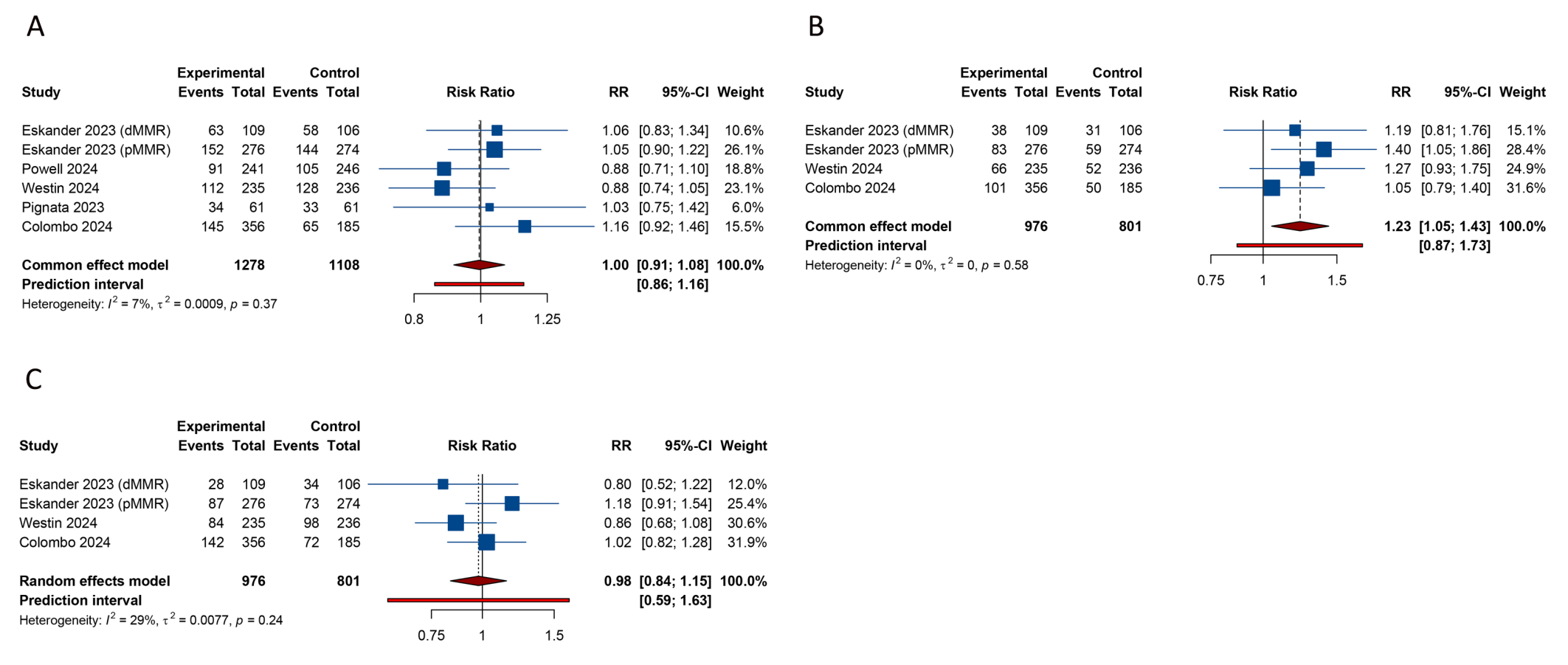


**FIGURE S14** Forest plots of any grade adverse events of gastrointestinal disorders. (A) Nausea; (B) Constipation; (C) Diarrhea; (D) Vomiting.


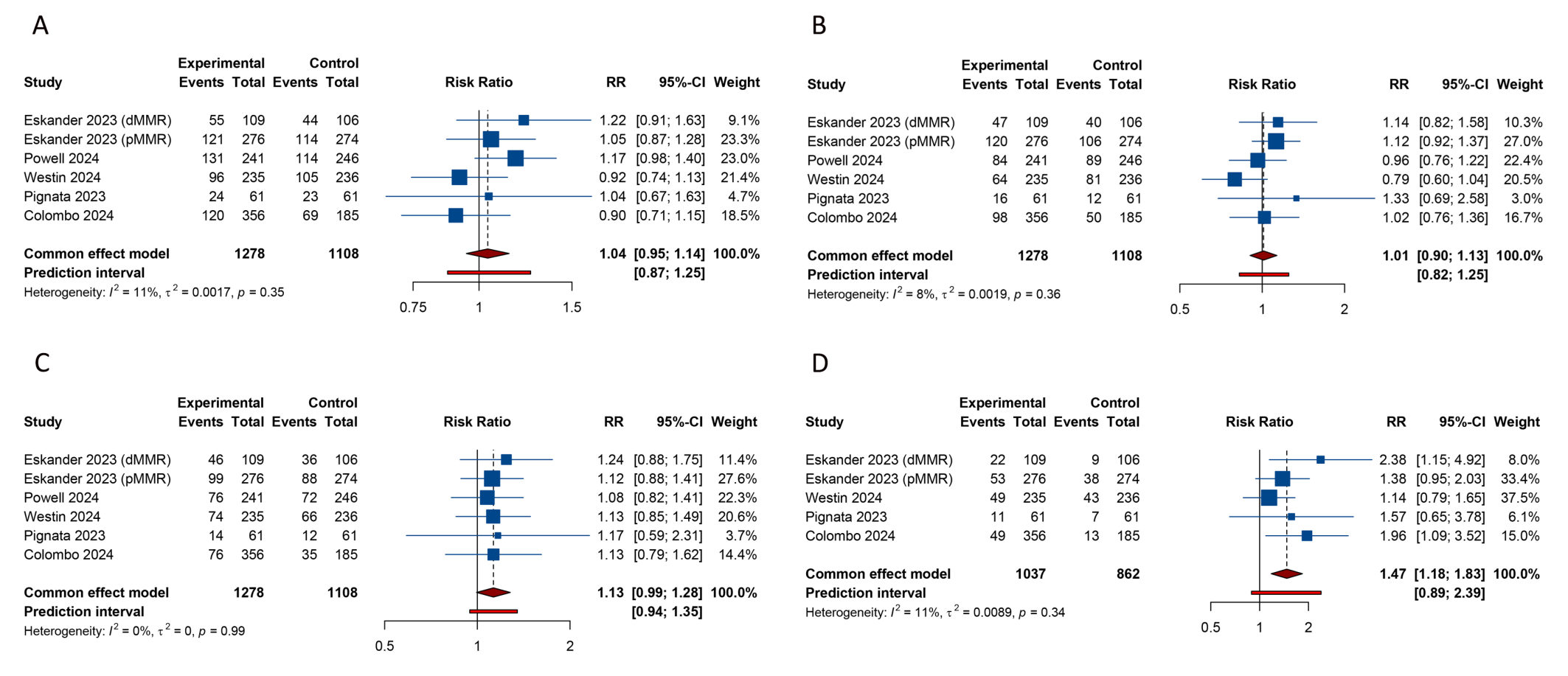


**FIGURE S15** Forest plots of any grade adverse events of musculoskeletal and connective tissue disorders. (A) Arthralgia; (B) Myalgia.


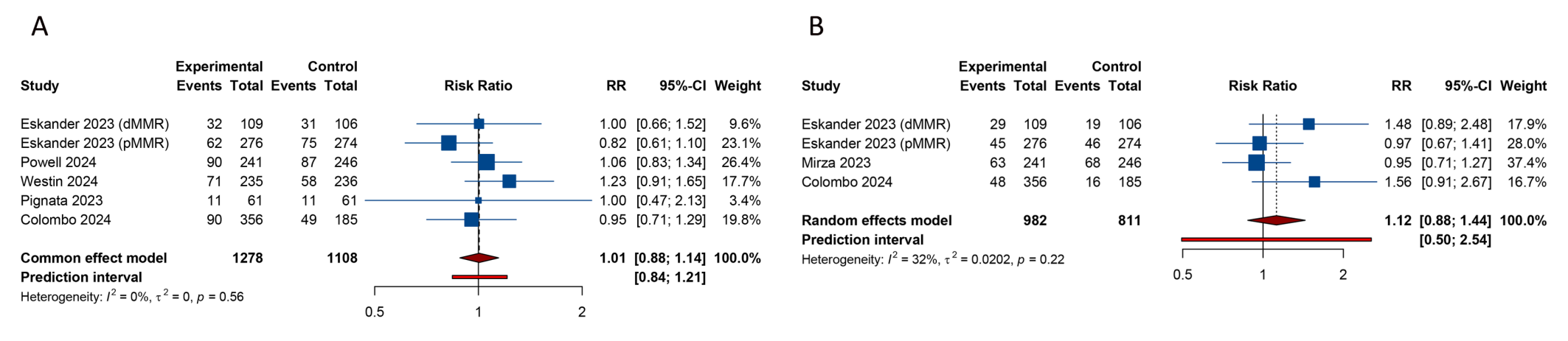


**FFIGURE S16** Forest plots of any grade adverse events of skin and subcutaneous tissue disorders. (A) Alopecia; (B) Rash.


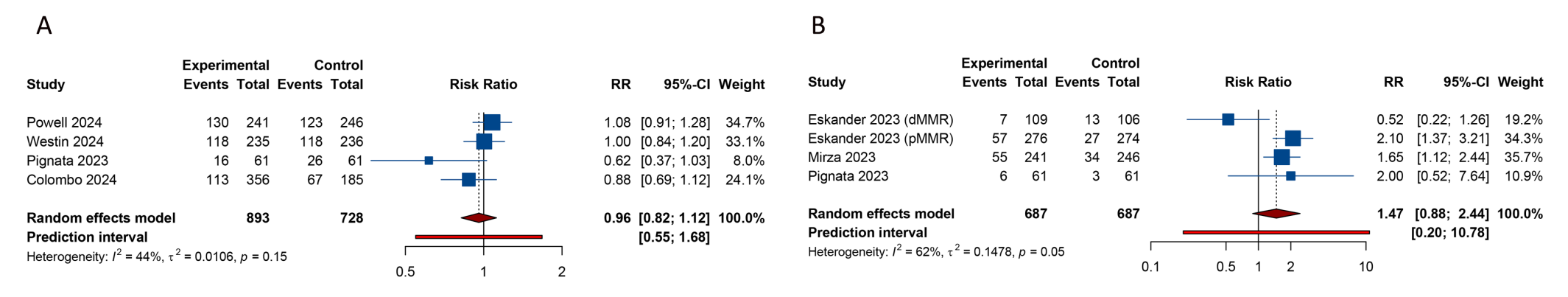


**FIGURE S17** Forest plots of any grade adverse events of other symptoms. (A) Fatigue; (B) Peripheral sensory neuropathy; (C) Dyspnea; (D) Decreased appetite; (E) Urinary tract infection.


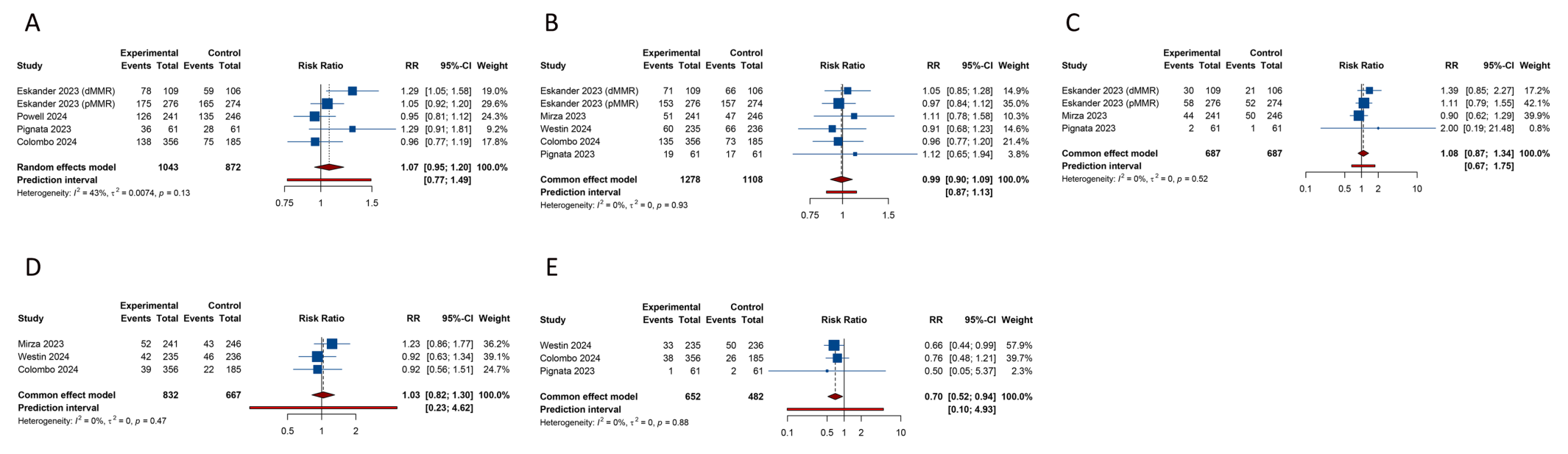


**FIGURE S18** Forest plots of grade ≥ 3 adverse events of blood and lymphatic system disorders. (A) Anemia; (B) Thrombocytopenia; (C) Neutropenia.


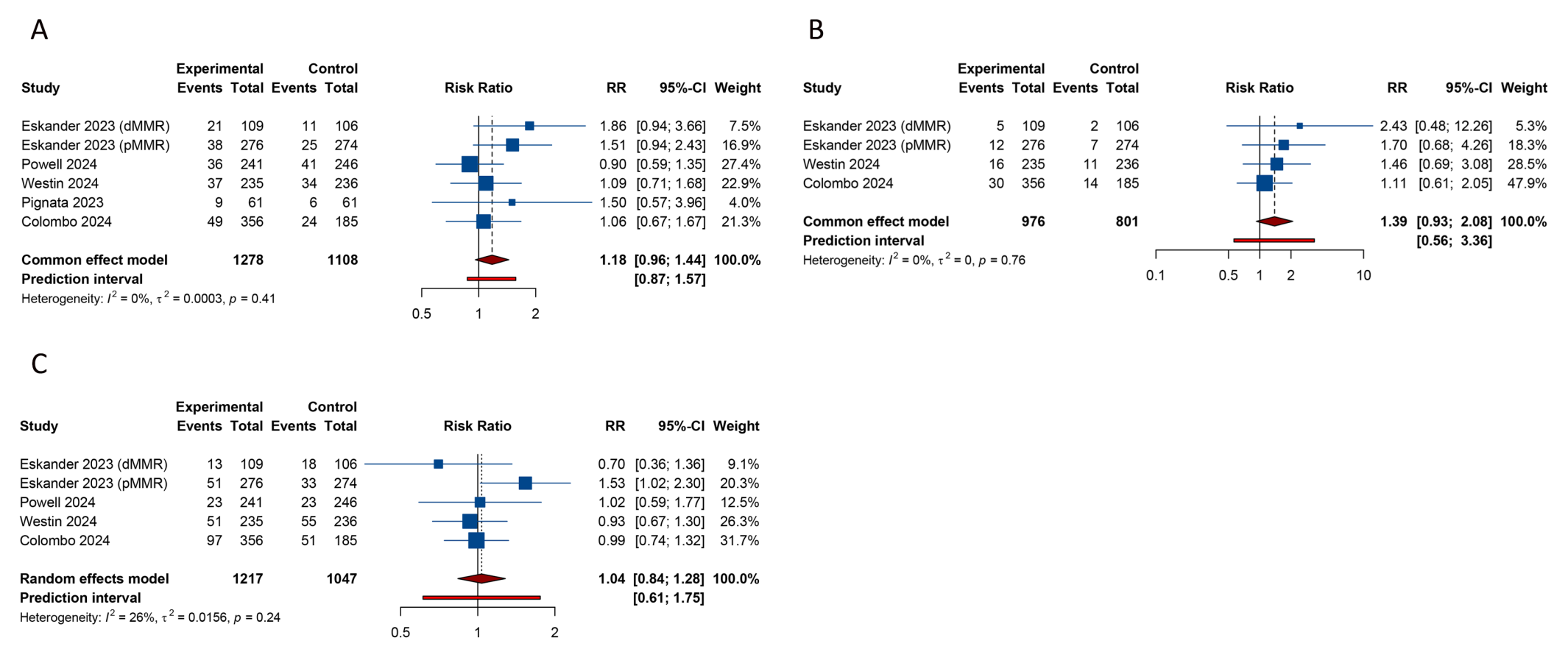


**FFIGURE S19** Forest plots of grade ≥ 3 adverse events of gastrointestinal disorders. (A) Nausea; (B) Constipation; (C) Diarrhea; (D) Vomiting.


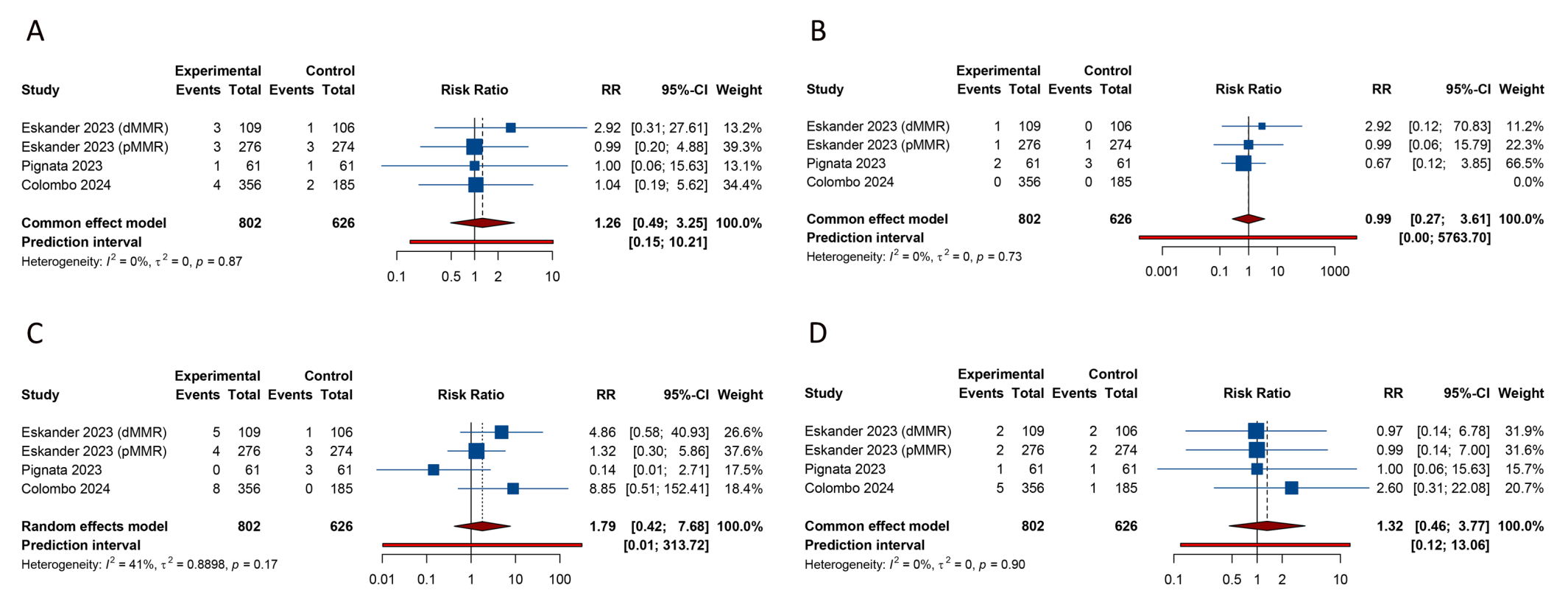


**FIGURE S20** Forest plots of grade ≥ 3 adverse events of other symptoms. (A) Fatigue; (B) Peripheral sensory neuropathy; (C) Arthralgia; (D) Hypertension.


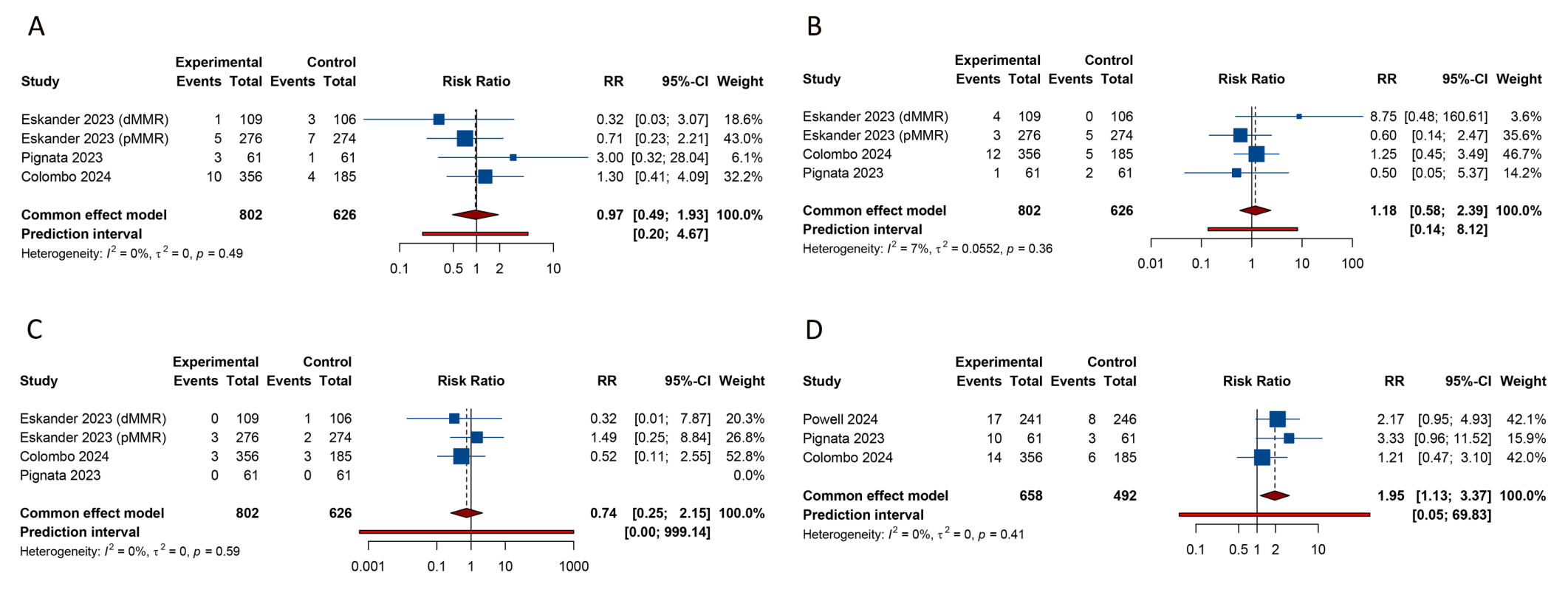


**FFIGURE S21** Sensitivity analysis of PD-1/PD-L1 inhibitors combined with chemotherapy for advanced or recurrent endometrial cancer. (A) Progression-free survival; (B) Overall survival; (C) Any grade adverse events; (D) Grade ≥ 3 adverse events.


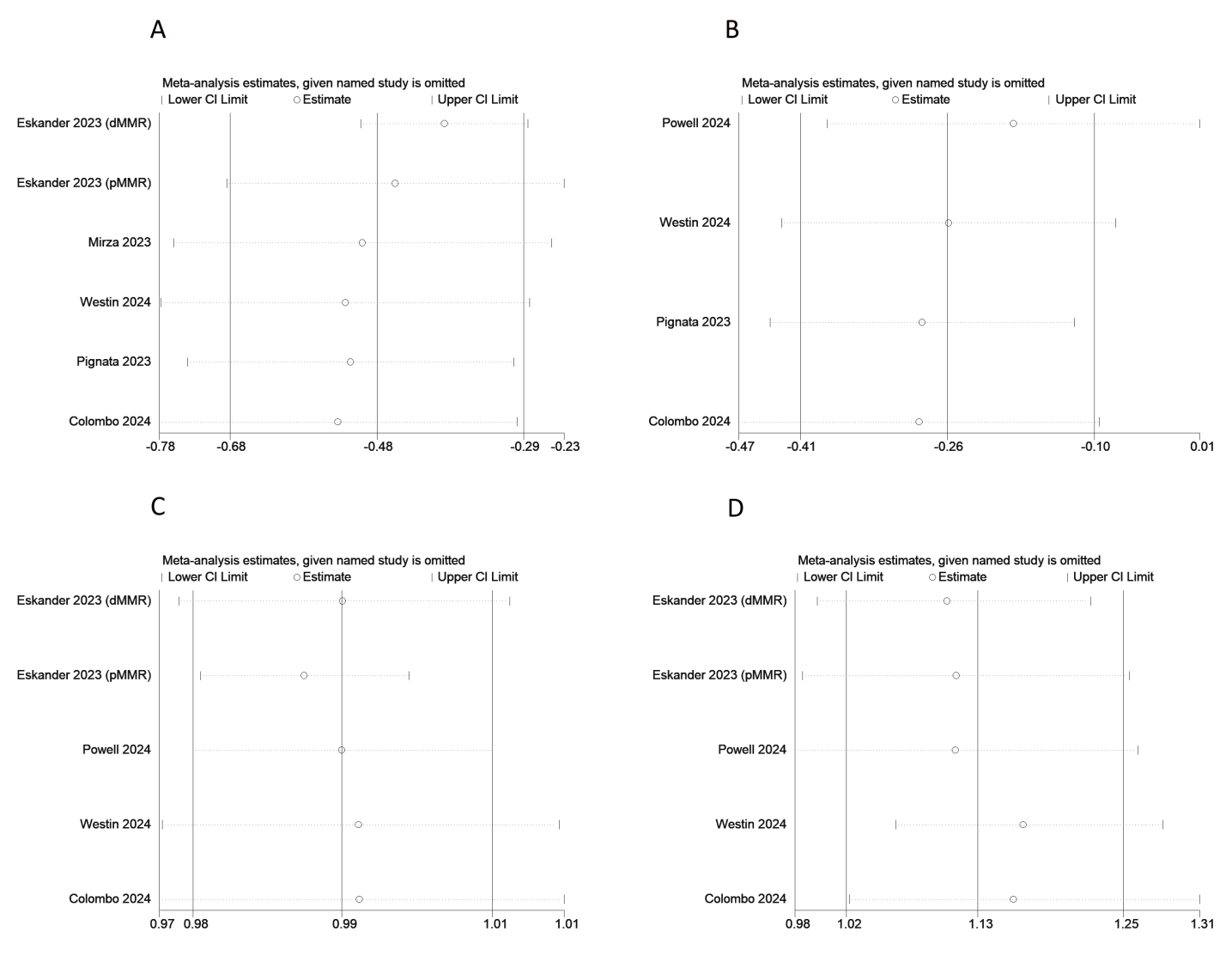


**FIGURE S22** Funnel plots of PD-1/PD-L1 inhibitors combined with chemotherapy for advanced or recurrent endometrial cancer. (A) Progression-free survival; (B) Overall survival; (C) Any grade adverse events; (D) Grade ≥ 3 adverse events.


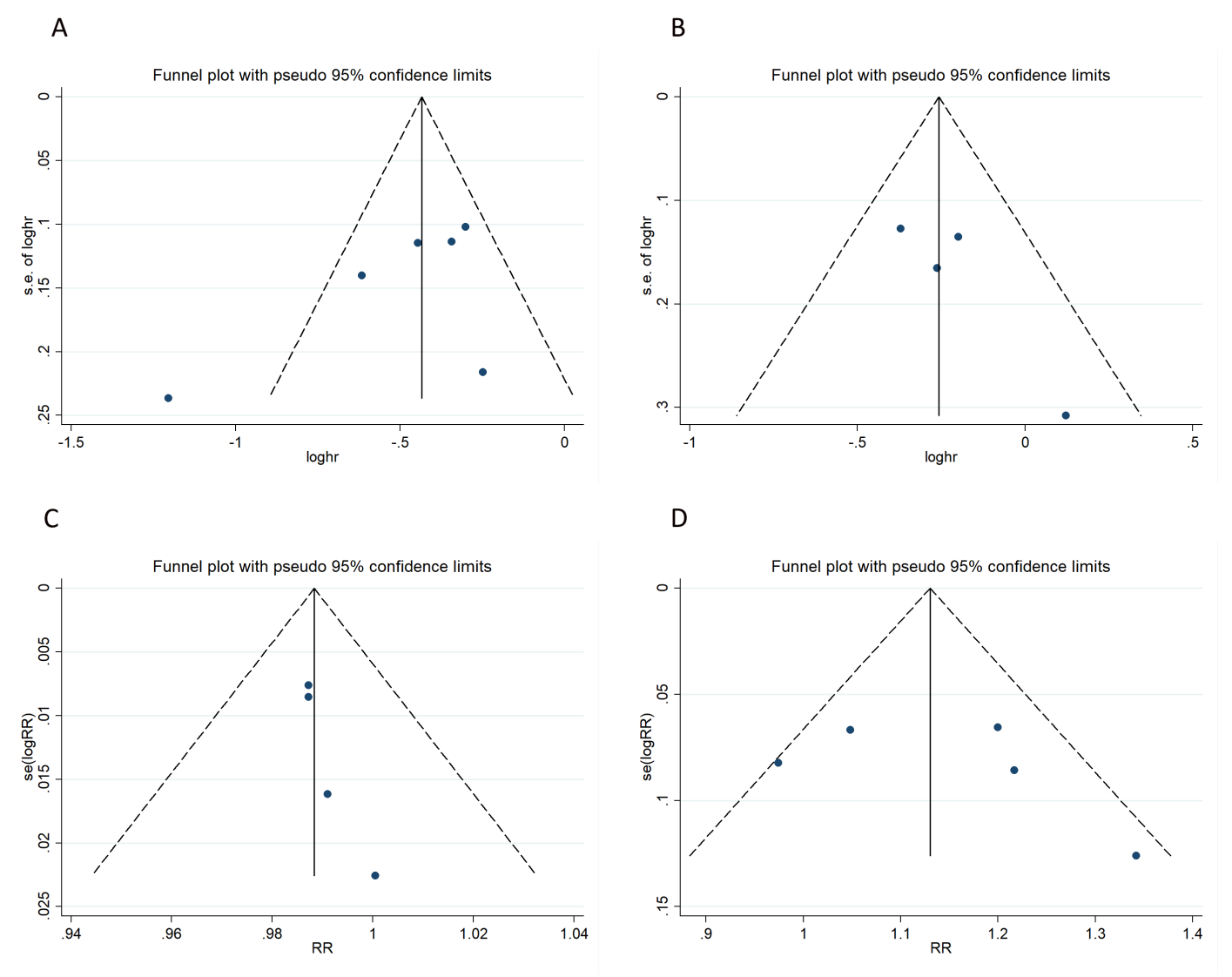

Supplement: Supplementary file 2 [file DataSheet2.docx]
