# Supplementary material for: Efficacy and safety of PD-1/PD-L1 inhibitors in advanced or recurrent endometrial cancer: a meta-analysis with trial sequential analysis of randomized controlled trials
Source: Front Immunol. 2025 Jan 31;16:1521362. doi: 10.3389/fimmu.2025.1521362 (PMC11825832; doi:10.3389/fimmu.2025.1521362)
Supplement: Supplementary file 4 [file Table1.docx]

| TABLE S1 Quality analysis of the included RCTs by modified Jadad scale. | | | | | | |
| --- | --- | --- | --- | --- | --- | --- |
| Study | Randomization | Randomization concealment | Double blind | Withdrawals and dropouts | Score | Study quality |
| Eskander, 2023 | 2 | 2 | 2 | 1 | 7 | High |
| Powell, 2024 | 2 | 2 | 2 | 1 | 7 | High |
| Westin, 2024 | 2 | 2 | 2 | 1 | 7 | High |
| Mirza, 2023 | 2 | 2 | 2 | 1 | 7 | High |
| Pignata, 2023 | 2 | 2 | 0 | 1 | 5 | High |
| Colombo, 2024 | 2 | 2 | 2 | 1 | 7 | High |
